# A small protein encoded by PCBP1-AS1 is identified as a key regulator of influenza virus replication via enhancing autophagy

**DOI:** 10.1371/journal.ppat.1012461

**Published:** 2024-08-13

**Authors:** Xiaojuan Chi, Guiying Huang, Liwei Wang, Xinge Zhang, Jiayin Liu, Zhihui Yin, Guijie Guo, Yuhai Chen, Song Wang, Ji-Long Chen

**Affiliations:** 1 Fujian Agriculture and Forestry University, Fuzhou, China; 2 Key Laboratory of Animal Pathogen Infection and Immunology of Fujian Province, College of Animal Sciences, Fujian Agriculture and Forestry University, Fuzhou, China; 3 Key Laboratory of Fujian-Taiwan Animal Pathogen Biology, College of Animal Sciences, Fujian Agriculture and Forestry University, Fuzhou, China; 4 CAS Key Laboratory of Pathogenic Microbiology and Immunology, Institute of Microbiology, Chinese Academy of Sciences, Beijing, China; Icahn School of Medicine at Mount Sinai, UNITED STATES OF AMERICA

## Abstract

Many annotated long noncoding RNAs (lncRNAs) contain small open reading frames (sORFs), some of which have been demonstrated to encode small proteins or micropeptides with fundamental biological importance. However, functions of lncRNAs-encoded small proteins or micropeptides in viral pathogenesis remain largely unexplored. Here, we identified a 110-amino acid small protein as a key regulator of influenza A virus (IAV) replication. This small protein that we call PESP was encoded by the putative lncRNA PCBP1-AS1. It was observed that both PCBP1-AS1 and PESP were significantly upregulated by IAV infection. Furthermore, they were markedly induced by treatment with either type I or type III interferon. Overexpression of either PCBP1-AS1 or PESP alone significantly enhanced IAV replication. In contrast, shRNA-mediated knockdown of PCBP1-AS1 or CRISPR/Cas9-mediated knockout of PESP markedly inhibited the viral production. Moreover, the targeted deletion or mutation of the sORF within the PCBP1-AS1 transcript, which resulted in the disruption of PESP expression, significantly diminished the capacity of PCBP1-AS1 to enhance IAV replication, underscoring the indispensable role of PESP in the facilitation of IAV replication by PCBP1-AS1. Interestingly, overexpression of PESP enhanced the IAV-induced autophagy by increasing the expression of ATG7, an essential autophagy effector enzyme. We also found that the 7–22 amino acids at the N-terminus of PESP were crucial for its functionality in modulating ATG7 expression and action as an enhancer of IAV replication. Additionally, HSP90AA1, a protein identified previously as a facilitator of autophagy, was found to interact with PESP, resulting in the stabilization of PESP and consequently an increase in the production of IAV. These data reveal a critical lncRNA-encoded small protein that is induced and exploited by IAV during its infection, and provide a significant insight into IAV-host interaction network.

## Author summary

Emerging evidence has shown that some lncRNAs encode biologically active small proteins or micropeptides involved in diverse cellular processes. Here, we identified a previously unrecognized small protein derived from the lncRNA PCBP1-AS1 and established a role for this protein in influenza A virus (IAV) infection. We named it as PCBP1-AS1-encoded small protein (PESP) and found it to be crucially implicated in replication of IAV. Expression of both PESP and PCBP1-AS1 were significantly increased in response to IAV infection or upon interferon treatment. By overexpressing PESP, we observed a substantial enhancement in IAV replication, which was counteracted by reducing PESP level. We further demonstrated that PESP coding sequences were essential for PCBP1-AS1 to facilitate IAV replication, as evidenced by significantly reduced functionality of PCBP1-AS1 with deletion of the sequences. Intriguingly, PESP appeared to promote viral replication by modulating autophagy. This was achieved by significant upregulation of ATG7, a key enzyme in autophagy. Additionally, we revealed that interaction of PESP with HSP90AA1, a protein known to support autophagy, contributed to the protein’s stability of PESP and consequently to increased IAV replication. This study sheds light on the intricate interplay between host factors and viral pathogens, offering potential targets for antiviral strategies.

## Introduction

Influenza A virus (IAV) is a segmented negative-strand RNA virus that can cause seasonal epidemics and occasional pandemics, posing a great threat to public health [1]. IAV invasion activates host innate immune responses by stimulating certain pattern recognition receptors (PRRs) including RIG-I, MDA5 and TLR3 [2,3]. The PRRs detect viral components, such as viral RNA, and trigger a signaling cascade, leading to the production of various cytokines, such as type I and type III interferons (IFNs). IFNs are key mediators of the innate immunity against IAV infection [4,5]. They upregulate the expression of hundreds of IFN-stimulated genes (ISGs), and induce an antiviral state in host, which inhibits viral replication and spread [6,7]. However, IAV has evolved multiple mechanisms to evade the host innate immunity. For example, the virus can inhibit the production of IFNs or block their signaling pathways by its NS1 protein [8,9]. IAV can also target and manipulate host proteins and non-coding RNAs (ncRNAs) involved in the antiviral immune responses [10]. In addition, IAV can exploit cellular machinery and various factors for its infection and replication, which makes successful adaptation to hosts [11].

Increasing studies have shown that IAV can manipulate the host immune response through regulation of long non-coding RNAs (lncRNAs), a class of ncRNAs that play important roles in many cellular processes [12–15]. However, aberrant expression of lncRNAs has been closely associated with the development of several diseases, such as cancer, autoimmune diseases, and viral pathogenesis [16–18]. In fact, many lncRNAs are significantly influenced by viral infection and involved in the host-virus interaction [14,19]. Some lncRNAs are manipulated by viruses to promote their pathogenesis, while others are part of the host’s defense mechanisms and are disadvantageous to viral survival. For instance, lncRNA NRAV negatively regulates antiviral innate immunity by affecting the histone modification of several critical ISGs, thereby repressing their initial transcription [20]. In contrast, lncRNA AVAN positively regulates the transcription of FOXO3a and binds directly to the E3 ligase TRIM25 to promote antiviral innate immunity, resulting in alleviated IAV virulence and decreased virus replication [21]. LncRNA CHROMR can shape active ISG promoters by binding to IRF-2 binding protein 2 (IRF2BP2), and functions in limiting viral infection of macrophages [22].

Recently, there are increasing evidences that some annotated lncRNAs containing small open reading frames (sORFs) encode functional micropeptides or small proteins. These micropeptides or small proteins are critical for different biological processes, including inflammatory response, muscle regeneration, apoptosis, metabolism, and tumorigenesis [23–25]. For example, the lncRNA MIR155HG encodes a 17-amino acid micropeptide that modulates antigen presentation by targeting HSC70 and suppresses autoimmune inflammation [26]. Another lncRNA specific to skeletal muscle encodes a conserved 46-amino acid micropeptide called MLN, which regulates physiological processes by inhibiting sarcoplasmic reticulum Ca^2+^-ATPase (SERCA) [27]. Moreover, a putative muscle-specific lncRNA encodes a 34-amino acid peptide named dwarf open reading frame (DWORF), which enhances SERCA activity in muscle [28]. A 94-amino acid micropeptide encoded by lncRNA LINC00467 is identified in colorectal cancer that promotes cancer progression by modulating ATP synthase activity [29]. LncRNA LOC90024 encodes a small 130-amino acid protein that facilitates "cancerous" RNA splicing and tumorigenesis [30]. Despite their importance, roles of lncRNAs-encoded micropeptides or small proteins are poorly understood in infectious diseases caused by viruses including IAV.

In this study, we identified a 110-amino acid small protein encoded by lncRNA PCBP1-AS1, which we named PCBP1-AS1 encoded small protein (PESP). We observed that both PCBP1-AS1 and PESP levels were significantly elevated by IAV infection and treatment with either type I or type III interferons. Functional analysis showed that overexpression of lncRNA PCBP1-AS1 or PESP significantly enhanced IAV replication, but mutation or deletion of PESP-encoding sequences in PCBP1-AS1 obviously impaired such function of the lncRNA. Consistently, silencing lncRNA PCBP1-AS1 or PESP also inhibited the IAV replication. Additionally, we demonstrated a regulation of ATG7 expression by PESP, and an interaction between PESP and HSP90AA1 that stabilized PESP and promoted autophagy, leading to increased viral production. These findings not only reveal endogenous existence of a small protein PESP, but also identify the IAV-induced PESP as a crucial regulator of the viral replication.

## Results

### Identification of an endogenous small protein encoded by lncRNA PCBP1-AS1

**Fig 1:**
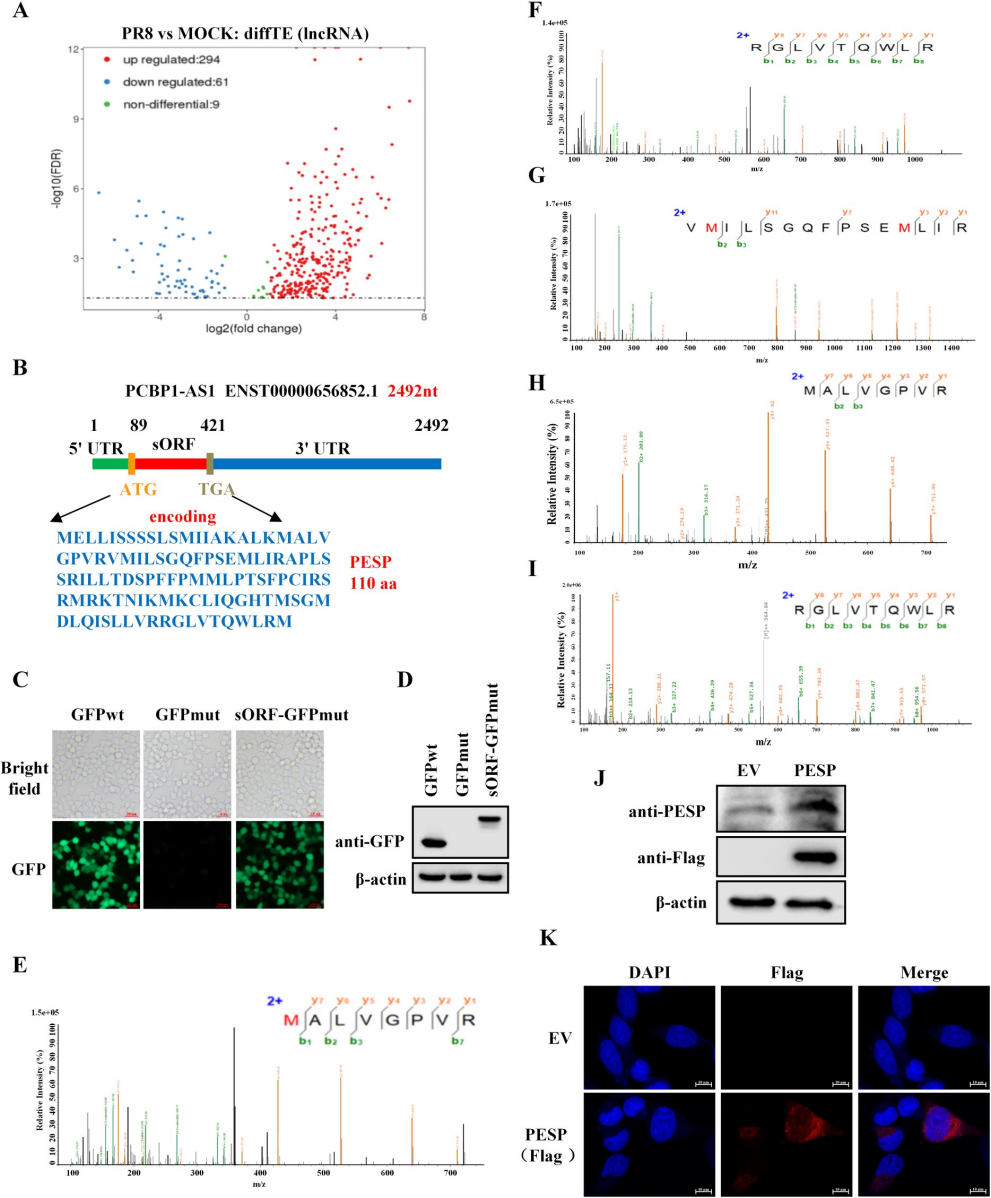
LncRNA PCBP1-AS1 encodes a 110-amino acid small protein called PESP. (A) A549 cells were infected with or without PR8 influenza virus for 12 h. Volcano plot showing differential translational efficiency (TE) analysis of lncRNAs between PR8-infected cells and control cells (log2 (fold change) > 1; false discovery rate < 0.05). (B) Schematic representation of lncRNA PCBP1-AS1 containing a potential small protein-encoding ORF, which may encode a 110-aa small protein, PESP. (C, D) The indicated constructs were transfected into 293T cells for 24h. GFP fluorescence was examined using an inverted fluorescence microscope (C), and protein expression was detected by Western blotting using an anti-GFP antibody (D). Scale bar, 100 μm. (E-G) Three unique peptides of PESP in A549 cells were identified using mass spectrometry. (H, I) MS/MS spectra of synthetic peptides that are identical to endogenous peptides. (J, K) The empty vector (EV), and PESP constructs fused with a Flag tag were transfected into 293T cells (J) and HeLa cells (K), and PESP-Flag expression were detected using anti-Flag and anti-PESP antibodies (J), and cellular localization of PESP was detected by immunofluorescence using anti-Flag antibody (K). Scale bar, 10 μm.

To identify functional involvement of small proteins or micropeptides encoded by lncRNAs in IAV pathogenesis, we performed several experiments including Ribo-seq (GSE252920) and RNA-seq (GSE252713) to determine translation ability of putative lncRNAs during the IAV infection. The integrative analysis of data sets revealed that among the lncRNAs, PCBP1-AS1 was bound by the ribosome, and its translation efficiency (TE) was clearly upregulated upon PR8 influenza virus infection (Fig 1A), suggesting that it may potentially encode a small protein or peptide. It was shown that there was a 333-nucleotide ORF with the potential to encode a 110-amino acid small protein in PCBP1-AS1 through Ribo-seq and bioinformatics analysis (Fig 1B). To confirm the potentiality of this sORF to encode a small protein, we employed a GFPmut ORF vector in which start codon ATGGTG of the pEGFP-N1 is mutated to ATTGTT. Thus, we generated a construct in which the sORF was fused to the N-terminus of the GFPmut ORF, and determined whether the sORF could drive the expression of GFP with mutant start codon. Indeed, the results displayed that start codon mutation abolished expression of GFP protein, but substantial expression of the sORF-GFP fusion protein was observed in cells transfected with the sORF-GFPmut vector (Fig 1C and 1D). Together, these observations suggest that the sORF in PCBP1-AS1 may encode a small protein, which we call PCBP1-AS1-encoded small protein (PESP).

Next, we asked whether there exists an endogenous PESP in cells. For this, cell lysates derived from A549 cell were separated by SDS-PAGE and examined by mass spectrometry (MS) analysis. Interestingly, three unique peptides contained in PESP sequences were detected in the cell lysates (Fig 1E–1G). To confirm this finding, we synthesized several peptides with same sequences as the identified peptides, and compared the MS spectra of the endogenous peptides with those of identical synthetic peptides (Fig 1H and 1I). The results exhibited a good spectral match between the endogenous peptides and the synthetic counterparts, providing strong evidence for the presence of PESP in the cells.

Moreover, we generated an anti-PESP antibody and performed immunoprecipitation, followed by analysis of mass spectrometry. The antibody detected well the PESP-Flag fusion protein by Western blotting (Fig 1J). Importantly, PESP-derived peptides were identified in the immunoprecipitate via mass spectrometry analysis, demonstrating that immunoprecipitate obtained from the prepared antibody contains endogenous PESP in A549 cells (S1A and S1B Fig). Additionally, cellular localization of PESP was investigated and it appeared to be primarily located in the cytoplasm (Fig 1K). Collectively, these observations indicate that PESP is naturally expressed in human cells and is predominantly distributed in the cytoplasm.

### PCBP1-AS1 and PESP are significantly induced by influenza virus infection

**Fig 2:**
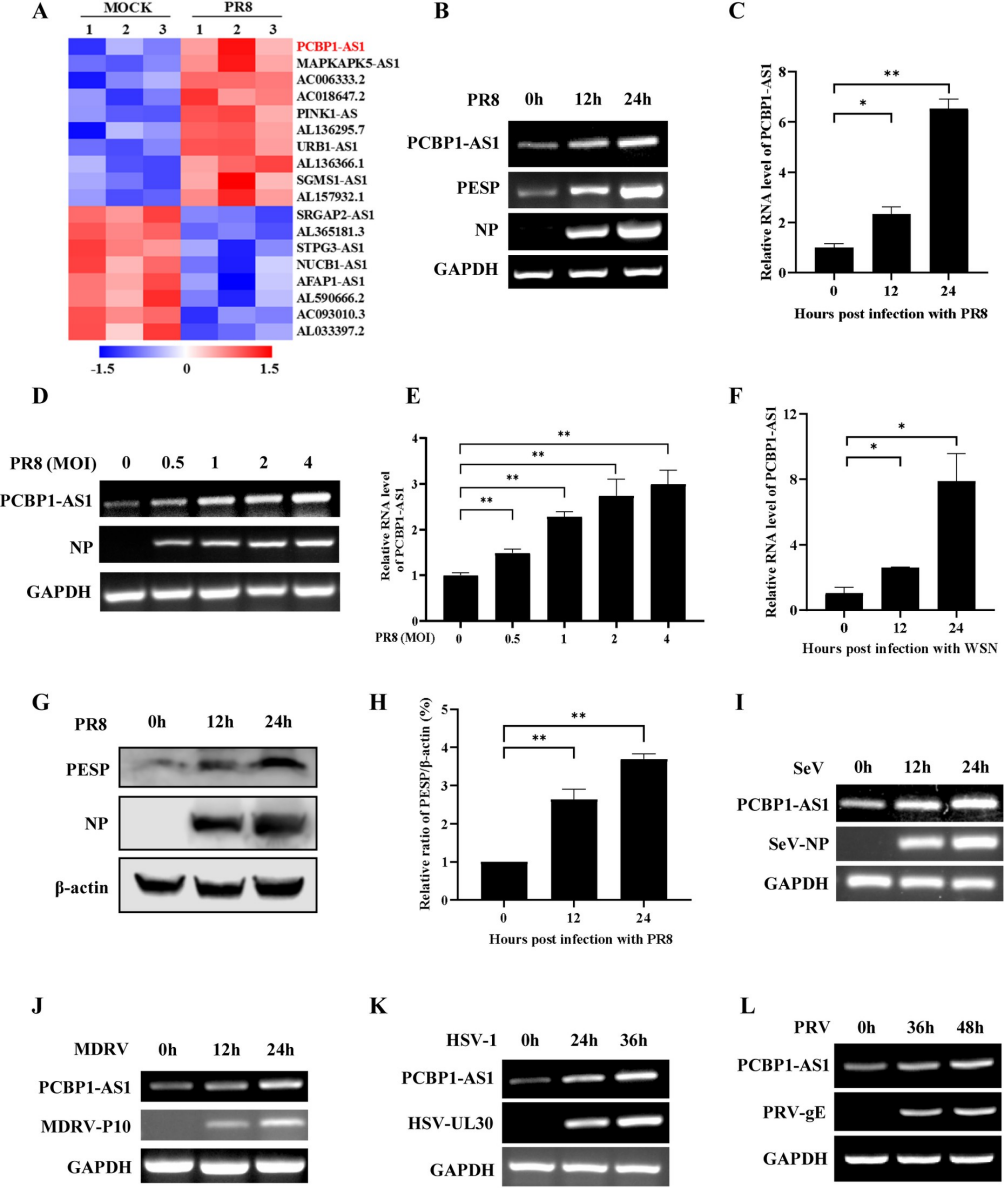
PCBP1-AS1 and PESP are significantly induced by viral infection. (A) The differentially expressed lncRNAs in A549 cells infected with or without PR8 influenza virus were analyzed by RNA-seq. Shown are representative differentially expressed lncRNAs. (B, C) The expression of PCBP1-AS1 in A549 cells infected with PR8 virus (MOI = 1) was detected by RT-PCR (B) and qRT-PCR (C). (D, E) A549 cells were infected with PR8 virus at indicated MOIs for 12 h. RT-PCR (D) and qRT-PCR (E) were performed to determine the expression of PCBP1-AS1. (F) A549 cells were infected with WSN virus (MOI = 1) for indicated hours, and then the expression of PCBP1-AS1 was detected by qRT-PCR. (G) The PESP expression in A549 cells infected with PR8 virus was detected by Western blotting. (H) Relative levels of PESP in (G) were quantitated by densitometry and normalized to β-actin levels. (I-L) The expression of PCBP1-AS1 in 293T cells infected with SeV (I), MDRV (J), HSV-1(K), or PRV (L) was examined by RT-PCR. Data are shown as means ± SD; n = 3; *p< 0.05, **p< 0.01.

RNA-seq data showed that expression level of PCBP1-AS1 was significantly elevated in A549 cells after IAV infection (Fig 2A). Further analysis by RT-PCR and qRT-PCR confirmed that the expression of PCBP1-AS1 was clearly upregulated in cells infected with PR8 virus in time-dependent and viral multiplicity of infection (MOI)-dependent manner (Fig 2B–2E). Similarly, the WSN influenza virus also induced a significant increase in the expression of PCBP1-AS1 at various time points post infection (Fig 2F). Next, we assessed the impact of IAV infection on PESP expression. Protein samples were collected from A549 cells infected with PR8 virus, and endogenous expression of PESP was then analyzed using the anti-PESP antibody. Consistently, PESP levels were also substantially upregulated upon PR8 virus infection (Fig 2G and 2H).

To investigate whether PCBP1-AS1 and PESP expression could be induced by infection with other viruses, we tested Sendai virus (SeV), Muscovy duck reovirus (MDRV), herpes simplex virus 1 (HSV-1), and pseudorabies virus (PRV). Interestingly, a significant increase in the expression of PCBP1-AS1 and PESP was observed in the cells infected with these viruses (Figs 2I–2L and S2), indicating a broad spectrum of PCBP1-AS1 and PESP induction by viral infections. Taken together, these data reveal that both PCBP1-AS1 and PESP are upregulated by IAV as well as other viral infections, suggesting their functional involvement in the interplay between these viruses and host.

### Expression of PCBP1-AS1 and PESP is regulated by interferon signaling

**Fig 3:**
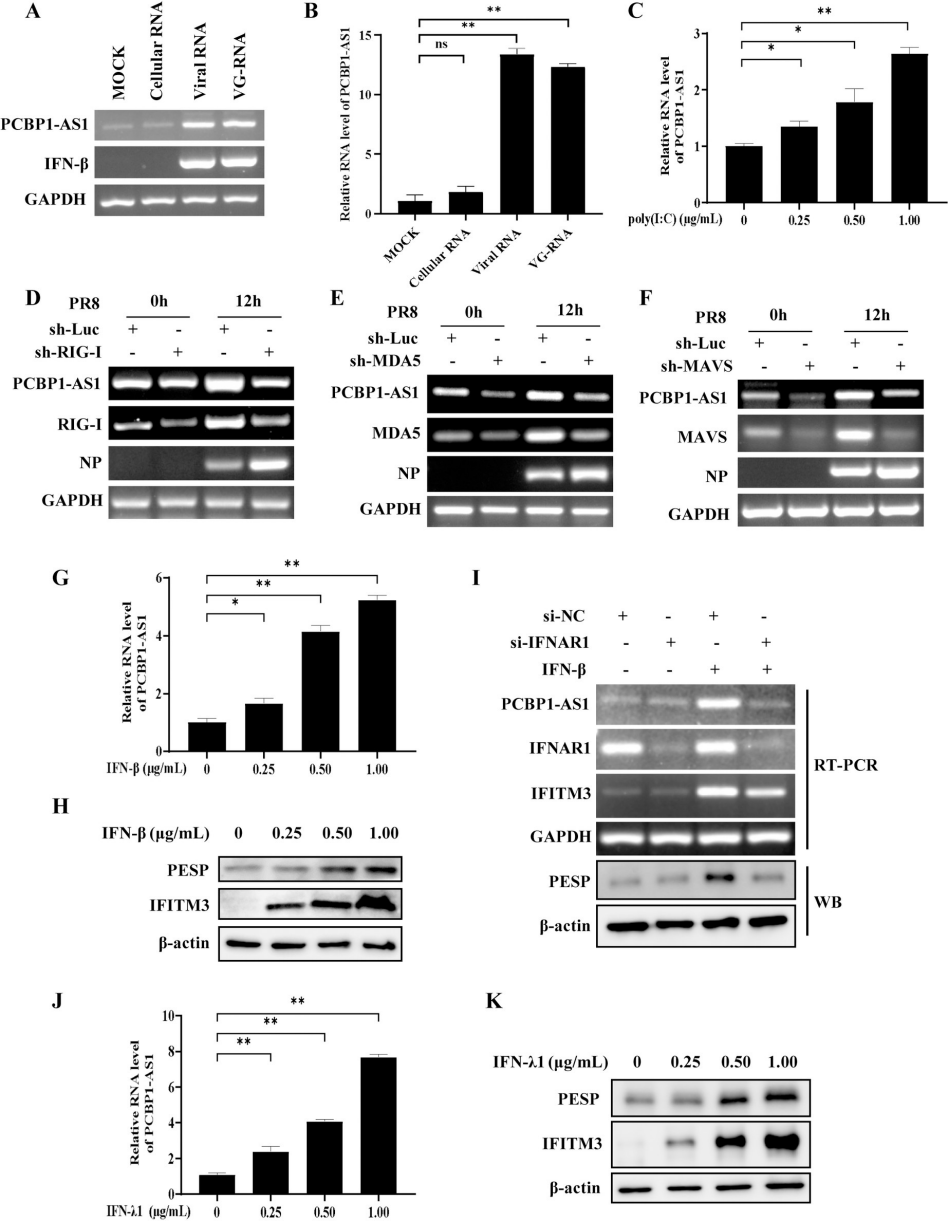
Expression of PCBP1-AS1 and PESP is regulated by interferon signaling. (A, B) The expression of PCBP1-AS1 in A549 cells transfected with viral RNA, VG-RNA, or cellular RNA for 8 h was detected by RT-PCR (A) and qRT-PCR (B). (C) A549 cells were transfected with various concentrations of poly(I:C) for 8 h. Then the cells were harvested, and the expression of PCBP1-AS1 was examined by qRT-PCR. (D-F) A549 cells stably expressing shRNA targeting RIG-I (D), MDA5 (E), MAVS (F) or luciferase control were infected with or without PR8 virus (MOI = 1) for 12 h. Total RNA was extracted and RT-PCR was performed to examine the expression of PCBP1-AS1. (G, H) A549 cells were treated with IFN-β at indicated concentrations for 6 h. The expression of PCBP1-AS1 was examined by qRT-PCR (G), and the expression of PESP was examined by Western blotting (H). (I) A549 cells were transfected with control siRNA or siRNA targeting IFNAR1. After 36 h of transfection, the cells were treated with or without IFN-β (1 μg/mL) for 6 h. Then the expression of PCBP1-AS1 and PESP was examined by RT-PCR and Western blotting, respectively. (J, K) A549 cells were treated with IFN-λ1 at indicated concentrations for 6 h. The expression of PCBP1-AS1 was examined by qRT-PCR (J), and the expression of PESP was examined by Western blotting (K). Data are shown as means ± SD; n = 3; *p< 0.05, **p< 0.01, and ns represents no significance.

We next determined how expression of PCBP1-AS1 and PESP was regulated by infection of the influenza virus. First, we transfected A549 cells with total RNA derivated from cells infected with the IAV for 24 hours (viral RNA), IAV genomic RNA (VG-RNA), and control RNA from uninfected cells (cellular RNA), respectively. Strikingly, an increase in the expression of PCBP1-AS1 was observed in cells transfected with either viral RNA or VG-RNA as compared to the cellular RNA (control) treatment group (Fig 3A and 3B). Second, we stimulated the cells with poly(I:C), a synthetic analogue of viral double-stranded RNA, and found that poly(I:C) treatment resulted in a concentration-dependent upregulation of PCBP1-AS1 expression (Figs 3C and S3A).

RIG-I and MDA5 are widely expressed cytosolic RNA helicases and critical for detecting RNA structures of influenza virus. Upon recognizing the invasion of the influenza virus, they become activated and bind to the signaling adaptor MAVS, triggering the activation of the interferon response [2–5]. Therefore, we examined the involvement of RIG-I, MDA5, and MAVS in regulating the IAV-induced expression of PCBP1-AS1. For this, we knocked down RIG-I, MDA5 or MAVS in A549 cells to disrupt innate immune signal transduction. Indeed, levels of PCBP1-AS1 were significantly decreased in these cells with impaired innate immune signaling as compared to the control cells (Fig 3D–3F).

It is well known that both viral RNA and poly(I:C) are inducers of type I and type III interferons, and the innate immune signaling pathway described above regulates interferon production. Finally, we determined whether the upregulation of PCBP1-AS1 upon viral infection was associated with increased expression of type I and type III interferons. As expected, stimulation with type I interferon IFN-β significantly enhanced the expression of PCBP1-AS1 in A549 cells (Figs 3G and S3B). PESP expression level was also substantially upregulated by the IFN-β treatment (Figs 3H and S3C). Silencing of the type I IFN receptor IFNAR1 using siRNA resulted in a significant reduction in IFN-β-induced expression of PCBP1-AS1 and PESP compared to control cells (Fig 3I). Similarly, treatment with type III interferon IFN-λ1 also upregulated the expression of PCBP1-AS1 and PESP (Figs 3J–3k and S3D). However, IL-6 and LPS treatment had no significant effect on the expression of PCBP1-AS1 (S3E and S3F Fig). These data indicate that both type I and type III interferons induce the expression of PCBP1-AS1 and PESP during the viral infection.

### Altering PCBP1-AS1 expression has a significant effect on IAV replication

**Fig 4:**
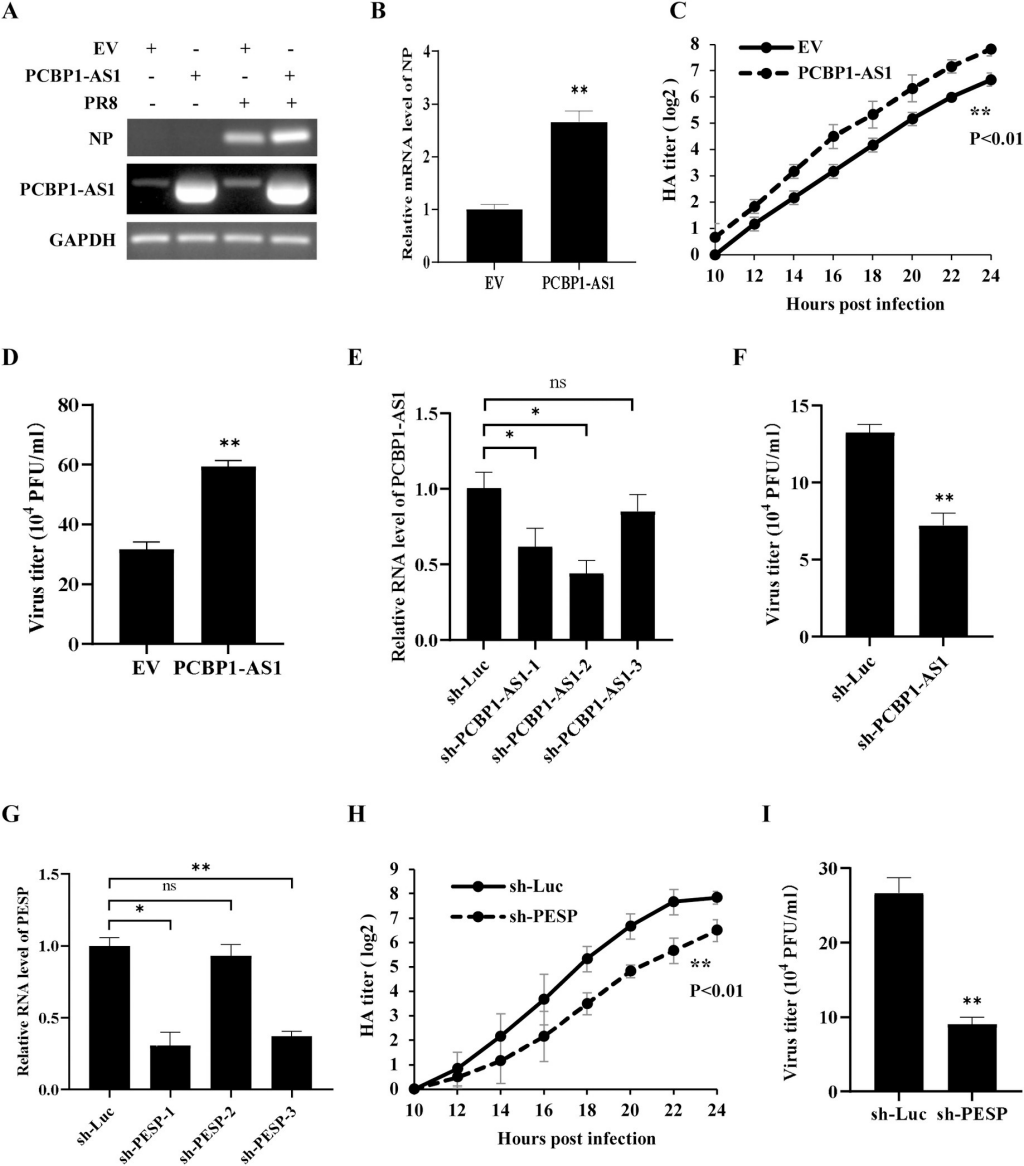
Altering PCBP1-AS1 expression has a significant effect on influenza virus replication. (A, B) A549 cells stably expressing PCBP1-AS1 or empty vector (EV) were infected with or without PR8 (MOI = 1) for 12 h. After infection, total RNA was extracted for RT-PCR (A) and qRT-PCR (B) to detect mRNA levels of viral NP. (C) The replication kinetics of PR8 virus (MOI = 1) in PCBP1-AS1-overexpressing A549 cells and control cells were examined by hemagglutinin (HA) assay. (D) PCBP1-AS1-overexpressing A549 cells and control cells were infected with PR8 virus (MOI = 1) for 16 h. Virus titers in culture supernatants were examined by plaque assay. (E) A549 cells stably expressing shRNA targeting the outside coding region of PESP in PCBP1-AS1 were generated, and the expression of PCBP1-AS1 in the cell lines was detected by qRT-PCR. The optimal knockdown effect was noted with sh-PCBP1-AS1-2, which was used in the subsequent experiments. (F) sh-PCBP1-AS1 cells and sh-Luc control cells were infected with PR8 virus (MOI = 1) for 16 h. Virus titers in the culture supernatants were examined by plaque assay. (G) A549 cells stably expressing shRNA targeting PESP were generated, and the expression of PESP in the cell lines was detected by qRT-PCR. The optimal knockdown effect was noted with sh-PESP-1, which was used in the subsequent experiments. (H, I) sh-PESP cells and sh-Luc control cells were infected with PR8 virus (MOI = 1). The culture supernatants were harvested at the indicated times for HA assay (H) and at 16 h for plaque assay (I) to measure virus titers. Data are represented as mean ± SD; n = 3; *p< 0.05, **p< 0.01, and ns represents no significance.

To explore the function of PCBP1-AS1 and its encoded small protein PESP in IAV infection, we constructed A549 cells stably expressing PCBP1-AS1. We found that overexpression of PCBP1-AS1 significantly increased the mRNA level of IAV NP (Fig 4A and 4B). Consistently, the viral replication was significantly enhanced by overexpression of PCBP1-AS1, as evidenced by the increased viral titers measured by hemagglutination assay and plaque-forming assay (Fig 4C and 4D).

On the other hand, we investigated the effects of PCBP1-AS1 knockdown on the viral replication by generating A549 cells that stably express specific shRNAs targeting different regions of PCBP1-AS1. The results showed that knockdown of PCBP1-AS1 using shRNA targeting the outside coding region of PESP (sh-PCBP1-AS1) significantly decreased viral titers in PR8-infected A549 cells compared to the control cells (Fig 4E and 4F). Similar results were obtained from the IAV-infected A549 cells in which PCBP1-AS1 was knocked down using shRNA targeting coding region of PESP (sh-PESP) (Fig 4G–4I). Collectively, these findings establish that PCBP1-AS1 enhances the replication of the influenza virus in A549 cells. However, it remains to be determined whether the lncRNA PCBP1-AS1 itself or its encoded small protein PESP is primarily responsible for promoting viral replication.

### PESP is essential for PCBP1-AS1 to facilitate IAV replication

**Fig 5:**
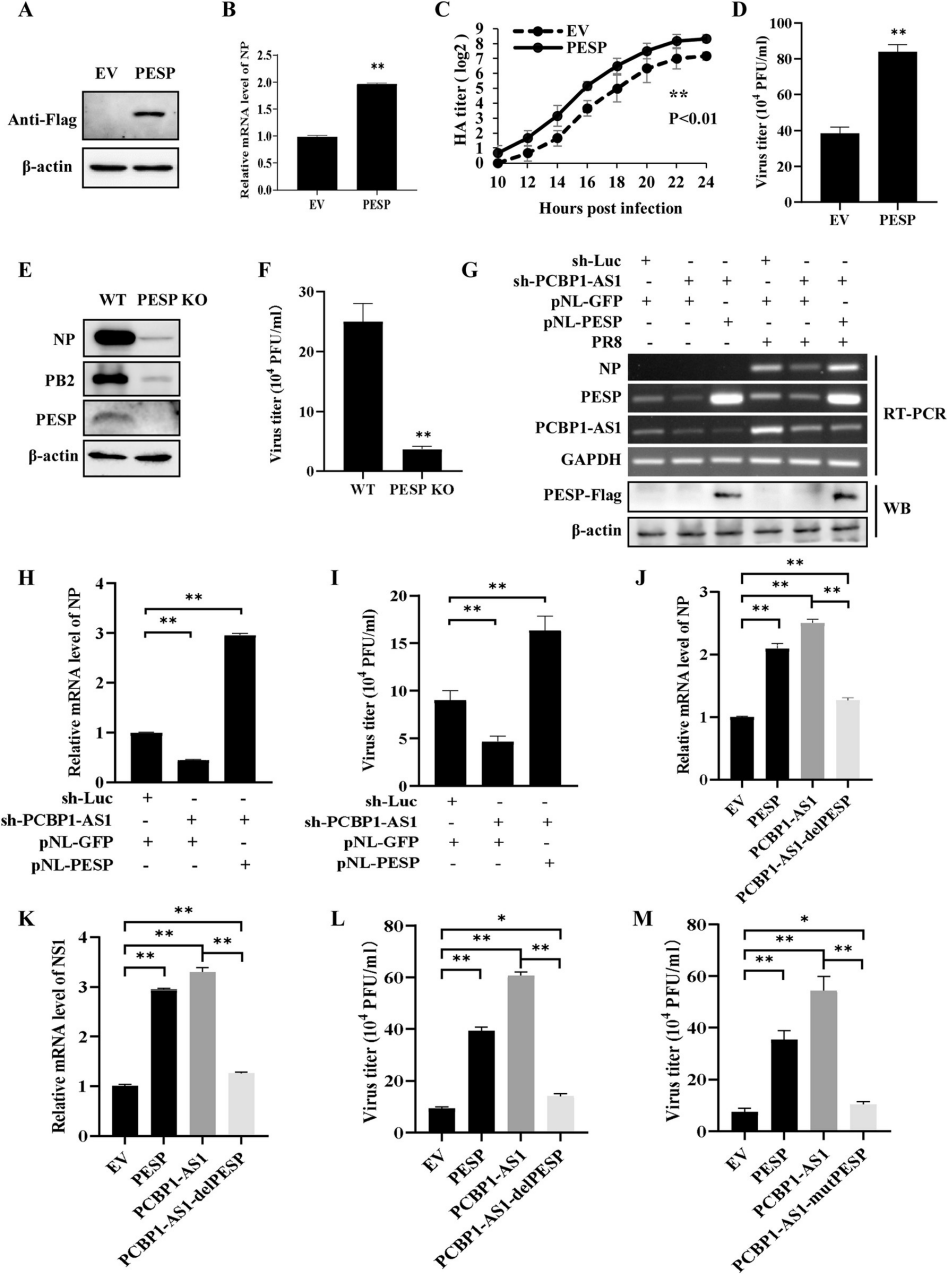
PESP is essential for PCBP1-AS1 to facilitate influenza virus replication. (A) A549 cells stably expressing PESP were generated, and the expression of PESP in the cell lines was determined by Western blotting. (B) The expression of viral NP in PESP-overexpressing cells and control cells infected with PR8 (MOI = 1) for 16 h was analyzed by qRT-PCR. (C, D) A549 cells overexpressing PESP and control cells were infected with PR8 virus (MOI = 1). The culture supernatants were harvested at the indicated times for HA assay (C) and at 16 h for plaque assay (D) to measure virus titers. (E, F) PESP knockout A549 cells and control cells were infected with PR8 (MOI = 1) for 16 h. The expression of viral NP and PB2 proteins was examined by Western blotting (E), and the viral titers in the supernatants of these cells were examined by plaque assay (F). (G-I) PCBP1-AS1 knockdown 293T cells and control cells were transfected with pNL-GFP or pNL-PESP, followed by infection with PR8 virus (MOI = 0.5) for 16 h. The mRNA levels of viral NP in the cells were examined by RT-PCR (G) and qRT-PCR (H), and the viral titers in the supernatants of these cells were examined by plaque assay (I). (J-L) 293T cells were transfected with EV, PESP, PCBP1-AS1 or PCBP1-AS1-delPESP, followed by infection with PR8 virus (MOI = 0.5) for 16 h. The mRNA levels of viral NP (J) and NS1 (K) in the cells were examined by qRT-PCR, and the viral titers in the supernatants of these cells were examined by plaque assay (L). (M) 293T cells were transfected with EV, PESP, PCBP1-AS1 or PCBP1-AS1-mutPESP, followed by infection with PR8 (MOI = 0.5) for 16 h. The viral titers in the supernatants of these cells were examined by plaque assay. Data are represented as mean ± SD; n = 3; *p< 0.05, **p< 0.01.

To examine directly the effect of PESP on IAV replication, PESP-overexpressing and empty vector (EV) control cell lines were generated and infected with PR8 virus. The results exhibited a significantly elevated level of the viral NP in PESP-overexpressing cells than that in control cells (Fig 5A and 5B). In line with this finding, forcing PESP expression resulted in a significant increase in viral titers measured by hemagglutination assay and plaque-forming assay (Fig 5C and 5D). To further validate the function of PESP in IAV replication, we generated PESP knockout (KO) A549 cells using CRISPR-Cas9 (S4A Fig). The CCK-8 cell proliferation assay revealed that PESP knockout did not impact the proliferation of A549 cells (S4B Fig). Notably, a dramatic decrease in viral NP and PB2 expression as well as viral titers was observed in the PESP KO cell as compared with the control cell infected with IAV (Fig 5E and 5F), demonstrating that PESP plays a critical role in IAV replication.

Moreover, we performed a rescue assay in PCBP1-AS1 knockdown cells. The expression of PCBP1-AS1 was disrupted in 293T cells via a lentiviral vector carrying shRNA targeting PCBP1-AS1. Consistent with our previous observation, a clear reduction in the viral NP expression and viral titers was found in PCBP1-AS1 knockdown cells (Fig 5G–5I). Interestingly, when PCBP1-AS1 knockdown cells were transfected with PESP-expressing plasmids, the expression level of NP and viral titers were not only restored but even much higher than those in PCBP1-AS1 knockdown and control cells (Fig 5G–5I). Together, these data suggest that PESP has a strong effect on the IAV replication.

To further confirm the implication of PESP in the IAV replication, we constructed several vectors expressing either PESP, PCBP1-AS1, PCBP1-AS1 lacking the coding region of PESP (PCBP1-AS1-delPESP), or PCBP1-AS1 with mutant start codon of PESP (PCBP1-AS1-mutPESP), and used EV as a control. 293T cells were then transfected with these plasmids, followed by infection with PR8 influenza virus. As expected, significant increases in viral NP and NS1 expression and viral titers were observed in cells overexpressing either PESP or PCBP1-AS1 compared to the EV control (Figs 5J–5L and S4C). However, viral titers and expression of viral NP and NS1 were significantly reduced in cells overexpressing PCBP1-AS1-delPESP compared to cells overexpressing PESP or PCBP1-AS1 after IAV infection (Figs 5J–5L and S4C). Similar results were obtained in the IAV-infected 293T cells expressing PCBP1-AS1 containing mutant PESP (PCBP1-AS1-mutPESP) (Figs 5M and S4D–S4E). Taken together, these observations imply that PCBP1-AS1 and its encoded PESP are critical for regulating IAV replication. Importantly, the results reveal that PESP is essential for PCBP1-AS1 to facilitate influenza virus replication.

### PESP promotes autophagy through upregulating the expression of ATG7

**Fig 6:**
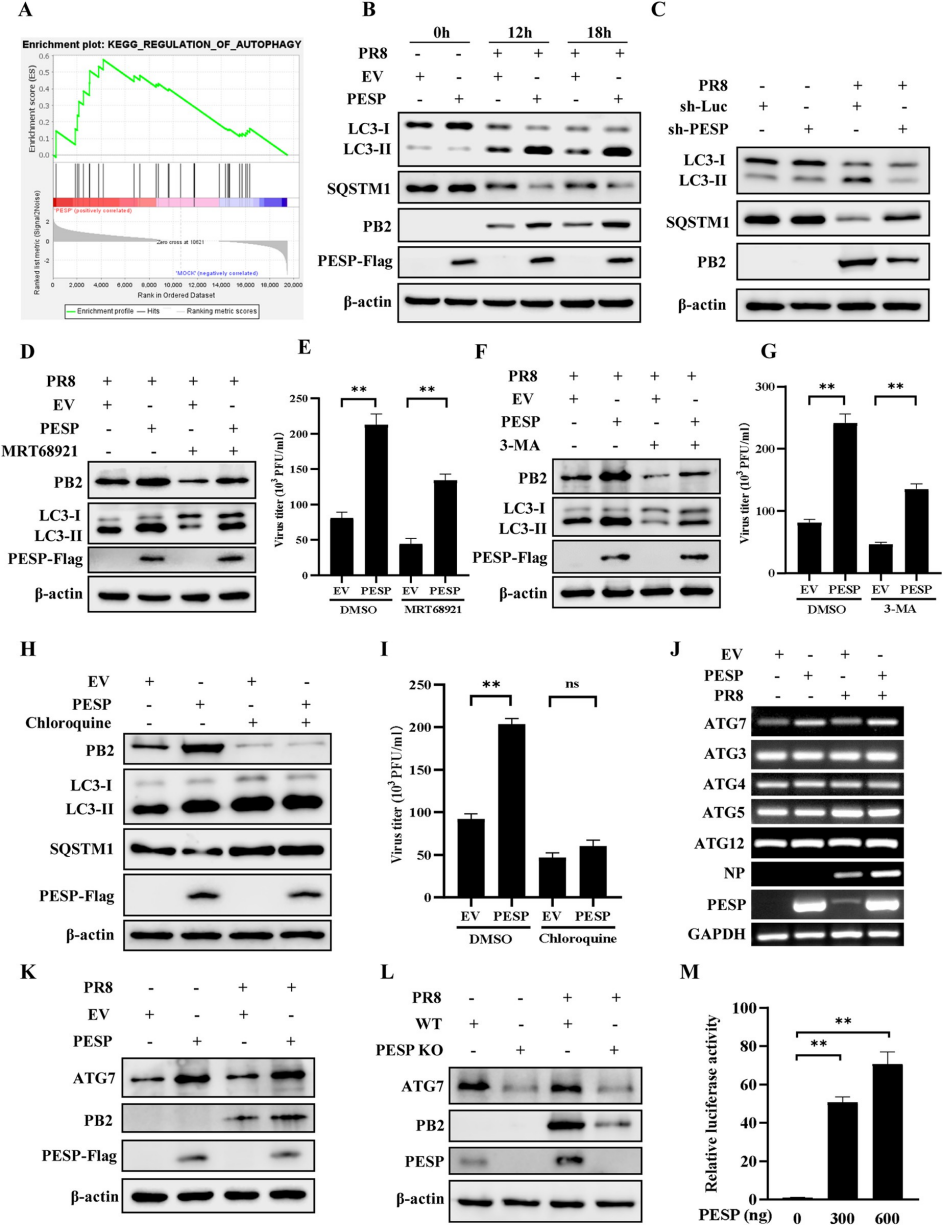
PESP promotes autophagy by upregulating the expression of ATG7. (A) GSEA enrichment plot of the regulation of autophagy pathway. (B) PESP-overexpressing A549 cells and control cells were infected with PR8 virus (MOI = 1) for the indicated times, and the protein levels of LC3 and SQSTM1 in the cells were determined by Western blotting. (C) PESP-knockdown A549 cells and control cells were infected with PR8 (MOI = 1) for 12 h. The levels of LC3 and SQSTM1 in the cells were examined by Western blotting. (D-I) PESP-overexpressing A549 cells and control cells were pretreated with 5 μM MRT68921 (D and E), 5 mM 3-MA (F and G), or 20 μM CQ (H and I) for 3 h, and then infected with PR8 virus (MOI = 1) for 12 h. The cells were harvested and analyzed by Western blotting with the indicated antibodies (D, F and H). Viral titers in the supernatants of these cells were examined by plaque assay (E, G and I). (J) PESP-overexpressing A549 cells and control cells were infected with PR8 (MOI = 1) for 12 h, and then the mRNA levels of indicated genes were detected by RT-PCR. (K, L) PESP-overexpressing (K) or PESP-knockout (L) A549 cells and control cells were infected with PR8 as described in (J). ATG7 protein level in the cells was determined by Western blotting. (M) The luciferase reporter plasmid containing ATG7 promoter (300 ng) was co-transfected with pRL-TK (30 ng), and PESP-overexpressing plasmid (300 ng and 600 ng) in 293T cells. After 36 h transfection, the cells were collected, and luciferase activities were detected. Data are represented as mean ± SD; n = 3; **p< 0.01, and ns represents no significance.

Next, we sought to gain an insight into the mechanism underlying regulation of influenza virus infection and replication by PESP. For this, we performed mRNA sequencing (GSE253139) on PESP-overexpression A549 cells and control cells infected with PR8 virus. Results obtained from Gene Set Enrichment Analysis (GSEA) revealed that ’autophagy’ was one of the most important pathways among significantly enriched KEGG in PESP-overexpression cells, indicating involvement of PESP in the IAV-induced autophagy (Fig 6A). To validate this finding, we examined the protein expression of autophagy-related marker LC3 and sequestosome 1 (SQSTM1) by Western blotting, and observed that overexpression of PESP caused a significant increase in the level of LC3-II and promoted the degradation of SQSTM1 compared with that in control cells (Fig 6B). When PESP was knocked down using shRNA in A549 cells, the accumulation of LC3-II and the degradation of SQSTM1 were obviously decreased compared to sh-Luc control cells upon challenge with IAV (Fig 6C). These data imply that PESP can promote autophagy and activate a complete autophagic response.

To further determine how PESP was involved in the IAV-induced autophagy, A549 cells overexpressing PESP or EV control were treated with either early-stage autophagy inhibitors MRT68921 and 3-methyladenine (3-MA), or late-stage autophagy inhibitor chloroquine (CQ). The experiments demonstrated that overexpression of PESP in the cells still significantly increased viral titers compared to control cells after treatment with MRT68921 or 3-MA (Fig 6D–6G). However, there were no significant differences in viral titers between the PESP-overexpressing group and control group treated with CQ (Fig 6H and 6I). These data suggest that PESP may function in regulating the elongation or maturation stages of autophagy.

We then examined the effect of PESP on the expression of several critical ATGs involved in autophagy elongation, such as ATG3, ATG4, ATG5, ATG7, and ATG12. Results from RT-PCR and Western blotting showed that the expression of ATG7, but not other ATGs, was significantly enhanced in PESP-overexpressing cells compared to control cells, while knockout of PESP in cells significantly decreased ATG7 expression (Figs 6J–6L and S5). Since PESP had the effects on expression of ATG7 at mRNA and protein levels, we investigated whether PESP could regulate the transcription of ATG7. To this end, we cloned the ATG7 promoter (from −1398 to −227) into a firefly luciferase vector and co-transfected it with PESP or an empty vector in 293T cells. Indeed, the dual-luciferase assay revealed a significant enhancement of ATG7 promoter activity by PESP (Fig 6M). Together, these findings suggest that PESP promotes IAV-induced autophagy through enhancing ATG7 promoter activity and thereby upregulating the expression of ATG7, which contributes to the increased replication of influenza virus [31].

### Identification of functional moieties in PESP

**Fig 7:**
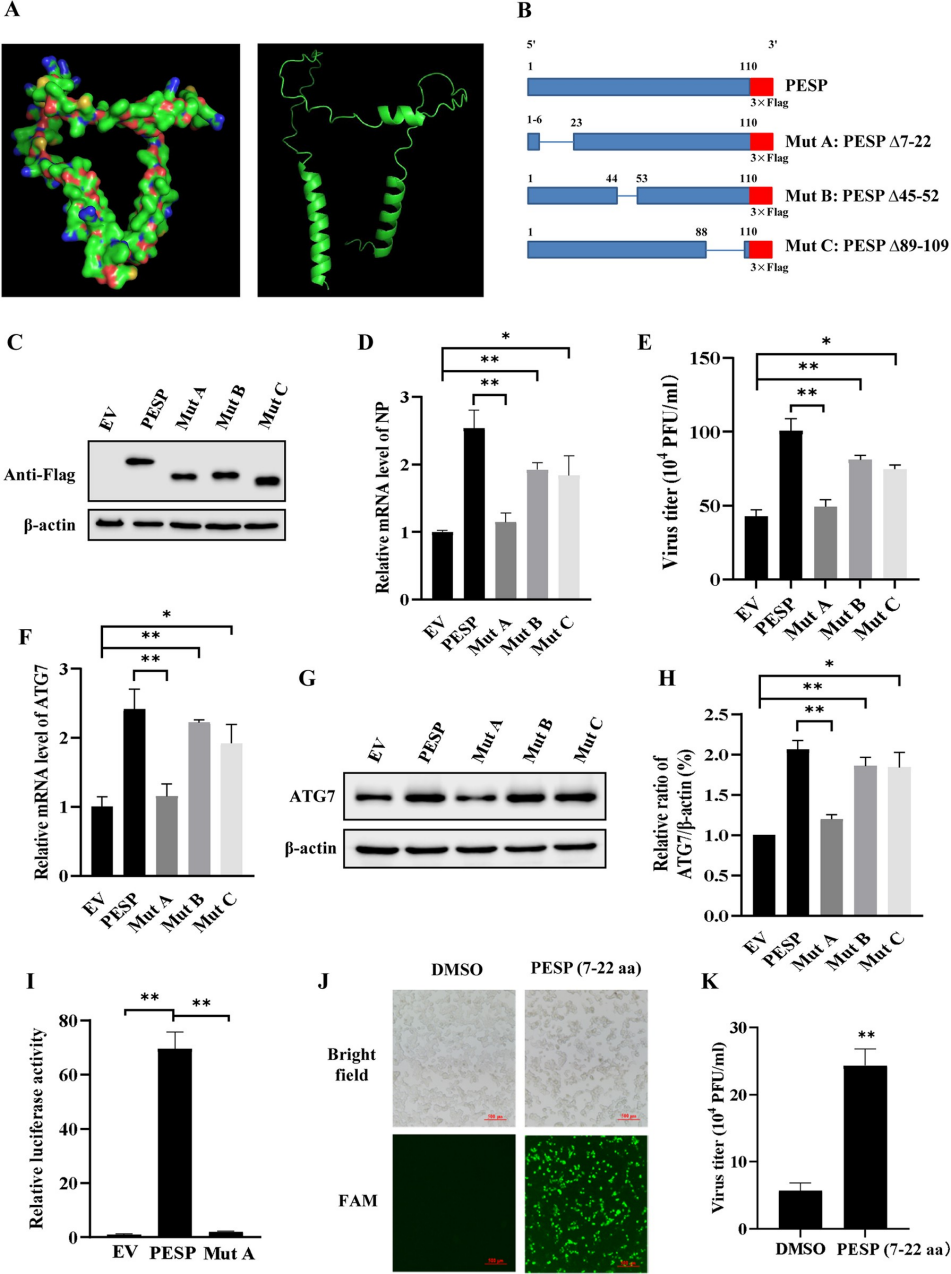
Identification of functional moieties that are required for PESP functioning. (A) The protein structure of PESP was predicted using AlphaFold2. (B) Schematic diagram of Flag-tagged deletion mutants of PESP. (C) The protein expression of wild-type and deletion mutants of PESP transfected into 293T cells was determined by Western blotting. (D, E) 293T cells expressing wild-type or deletion mutants of PESP were infected with PR8 (MOI = 0.5) for 16 h. The mRNA levels of viral NP were detected by qRT-PCR (D), and viral titers in the culture supernatants were determined by plaque assay (E). (F-H) 293T cells expressing wild-type or deletion mutants of PESP were infected with PR8 as described in (D) and (E). ATG7 expression in the cells was detected by qRT-PCR (F) and Western blotting (G). Relative levels of ATG7 in (G) were quantitated by densitometry and normalized to β-actin levels (H). (I) The luciferase reporter plasmid containing ATG7 promoter (300 ng) was co-transfected with pRL-TK (30 ng), PESP- or Mut A-overexpressing plasmid (600 ng) in 293T cells. After 36 h transfection, the cells were collected, and luciferase activities were detected. (J) Representative fluorescence microscopy images of 293T cells treated with DMSO or 10 μM FAM-conjugated PESP (7–22 aa) for 6 h. Scale bar, 500 μm. (K) 293T cells were treated with DMSO or 10 μM cell-permeable PESP (7–22 aa) for 6 h, and then infected with PR8 (MOI = 0.5) for 16 h. The viral titers in the supernatants of these cells were examined by plaque assay. Data are represented as mean ± SD; n = 3; *p< 0.05, **p< 0.01.

Since PESP is a newly found protein, we sought to identify domain(s) in the protein that are required for its functional involvement in regulating autophagy and IAV replication. To this end, AlphaFold2 analysis was employed to predict its structure. As shown in Fig 7A, the predicted structure of PESP is composed of three α-helices with varying lengths. The first α-helix consists of 16 residues (SSSLSMIIAKALKMAL), the second α-helix consists of 8 residues (LSSRILLT), and the third α-helix consists of 21 residues (SGMDLQISLLVRRGLVTQWLR). Furthermore, the entire peptide chain adopts a hairpin-like conformation, with the first and third helices positioned on opposite sides of the hairpin structure, and the second helix located at the apex of the hairpin.

To understand the function of these α-helices, we generated three mutants with different deletions in the α-helices and introduced a 3×Flag tag at the C-terminus of PESP (Fig 7B). The three deletion mutants, named Mut A (PESP Δ7–22), Mut B (PESP Δ45–52), and Mut C (PESP Δ89–109), were transfected into 293T cells, and the expected protein bands were detected by Western blotting using an anti-Flag antibody (Fig 7C). Notably, deletion of the α-helix in PESP impaired its ability to promote the IAV replication. Specifically, cells transfected with Mut A showed a marked decrease in the expression of viral NP and viral titers compared to cells expressing wild type PESP, comparable to those observed in EV-transfected control cells (Fig 7D and 7E). However, expression of Mut B and Mut C only caused a mild decrease in viral NP and viral titer compared to cells expressing wild type PESP, indicating that they still promoted the IAV replication to some extents (Fig 7D and 7E). Moreover, results from experiments using qRT-PCR and Western blotting showed that expression of ATG7 was significantly impaired in Mut A-expressing cell compared with that in PESP-expressing cell, while transfection with Mut B or Mut C in 293T cells had no significant effect on the expression of ATG7 (Fig 7F–7H). The dual-luciferase assay also demonstrated a significant decrease in ATG7 promoter activity induced by Mut A compared to the full-length PESP (Fig 7I).

To directly address the function of the first α-helix of PESP, we chemically synthesized FAM-conjugated peptide of PESP (7–22 aa) incorporating a cell-penetrating peptide (YGRKKRRQRRR). Our results showed that the synthetic PESP (7–22 aa) was able to penetrate 293T cells (Fig 7J), and treatment with PESP (7–22 aa) significantly increased viral titers as compared to the control group (Fig 7K). These experiments indicate that the α-helix (7–22 aa) at the N-terminus is a critical region for PESP functioning as an enhancer of IAV replication via regulation of ATG7 level.

### Interaction between PESP and HSP90AA1 increases its protein stability

**Fig 8:**
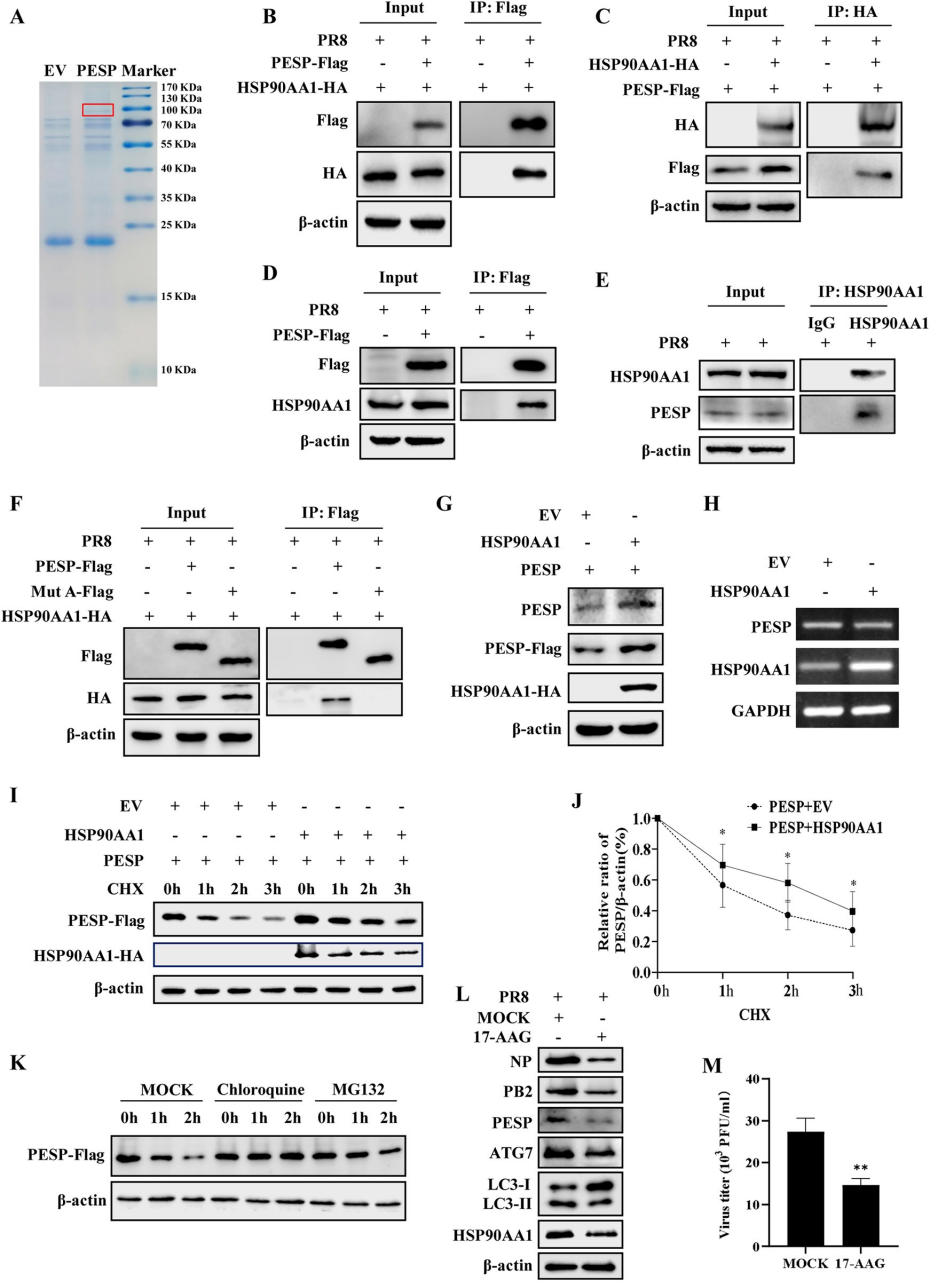
Interaction between PESP and HSP90AA1 enhances protein stability of PESP. (A) Proteins that interacted with PESP were identified by immunoprecipitation (IP). (B, C) 293T cells were co-transfected with PESP-Flag and HSP90AA1-HA plasmids, followed by infection with PR8 virus. PESP-Flag was immunoprecipitated from the lysates with anti-Flag antibody and the precipitates were blotted with anti-HA antibody (B). HSP90AA1-HA was immunoprecipitated from the lysates with anti-HA antibody and the precipitates were blotted with anti-Flag antibody (C). (D) 293T cells were transfected with or without PESP-Flag plasmids, followed by infection with PR8 virus. PESP-Flag was immunoprecipitated from the lysates with anti-Flag antibody and the precipitates were blotted with anti-HSP90AA1 antibody. (E) 293T cells were infected with PR8 virus, followed by immunoprecipitation with anti-HSP90AA1 antibody or IgG antibody as control, and the precipitates were blotted with anti-PESP antibody. (F) PESP-Flag or Mut A-Flag plasmid was transfected into 293T cells together with HSP90AA1-HA plasmid, followed by infection with PR8 virus. PESP-Flag and Mut A-Flag were immunoprecipitated from the lysates with anti-Flag antibody and the precipitates were blotted with anti-HA antibody. (G) 293T cells were co-transfected EV or HSP90AA1-HA plasmid together with PESP-Flag plasmid, and then the protein levels of PESP were determined by Western blotting using anti-PESP and anti-Flag antibodies. (H) 293T cells were transfected with EV or HSP90AA1-HA plasmid, and the mRNA levels of PESP were detected by RT-PCR. (I) 293T cells were co-transfected EV or HSP90AA1-HA plasmid together with PESP-Flag plasmid, followed by treatment with CHX (100 μg/mL). Cells were harvested at the indicated times and cell extracts were prepared for Western blotting to analyze PESP protein levels. (J) PESP levels in (I) were quantitated by densitometry and normalized to β-actin levels. (K) 293T cells transfected with PESP-Flag plasmid were treated with CHX (100 μg/mL), and then the cells were either MOCK-treated or treated with either CQ (20 μM) or MG132 (10 μM). At the indicated times, cells were harvested and analyzed by Western blotting with anti-Flag antibody. (L and M) 293T cells were pretreated with either DMSO or 17-AAG (50 nM) for 1 h, and then infected with PR8 virus for 12 h. The cells were harvested and analyzed by Western blotting with the indicated antibodies (L). The viral titers in the supernatants of the cells were examined by plaque assay (M). Data are represented as mean ± SD; n = 3; *p< 0.05, **p< 0.01.

Next, we asked whether PESP regulated autophagy and the viral replication through interaction with other protein(s). Thus, we performed an immunoprecipitation assay followed by mass spectrometry to identify such partners. When the immunoprecipitate was subjected to electrophoresis of SDS-PAGE, one specific band was obtained and then analyzed by mass spectrometry, which corresponded to HSP90AA1, a highly conserved molecular chaperone (Fig 8A). To confirm the interaction between PESP and HSP90AA1, several experiments were performed. First, we ectopically expressed both PESP-Flag and HSP90AA1-HA in 293T cells, and the cell lysates were immunoprecipitated by an anti-Flag antibody and probed with an anti-HA antibody. Indeed, PESP and HSP90AA1 were detected in the immunoprecipitate (Fig 8B). Second, similar results were obtained when immunoprecipitation was carried out by using an anti-HA antibody and probed with an anti-Flag antibody (Fig 8C). Third, immunoprecipitation experiment demonstrated that overexpressed PESP also interacted with endogenous HSP90AA1 (Fig 8D). Finally, immunoprecipitation using an anti-HSP90AA1 antibody revealed that endogenous PESP and HSP90AA1 bound to each other (Fig 8E). However, the Mut A with deletion of the 7–22 aa (PESP Δ7–22) lost its capacity to interact with HSP90AA1 (Fig 8F), indicating that the 7–22 amino acid region at the N-terminus of PESP is critical for mediating the interaction between PESP and HSP90AA1.

It is thought that peptides and small proteins encoded by ncRNAs are unstable and rapidly degraded in cells [32]. Interestingly, we found that protein level of PESP was clearly higher in HSP90AA1-overexpressing cells than that in control cells (Fig 8G). To determine whether the upregulation of PESP protein level was a result of an increase in its mRNA level, 293T cells were transfected with HSP90AA1-expressing plasmids or empty vector control and mRNA level of PESP was examined by RT-PCR. It was observed that overexpression of HSP90AA1 had little effect on PESP mRNA level (Fig 8H), indicating that the increase in PESP protein amount may be due to stability improvement through interaction with HSP90AA1. We further estimated the relative half-lives of PESP protein in cells with or without overexpression of HSP90AA1. The results displayed that half-life of PESP protein in cells with overexpression of HSP90AA1 was approximately 2-fold longer than that in cells without HSP90AA1 overexpression (Fig 8I and 8J). These data imply that HSP90AA1 increased the stability of PESP protein in the cells. To investigate the degradation pathway of PESP protein, cells transfected with PESP-expressing plasmids were co-treated with CHX and either the proteasome inhibitor MG132 or the lysosome inhibitor chloroquine. We found that treatment with chloroquine significantly slowed down the degradation rate of PESP protein, while treatment with MG132 had no significant effect on the stability of PESP (Fig 8K). Similarly, when HSP90AA1 was inhibited by 17-AAG, the endogenous PESP protein level was markedly decreased in 293T cells (Fig 8L). Consistent with this finding, treatment with 17-AAG resulted in the inhibition of autophagy, as indicated by decreased LC3-II and ATG7 (Fig 8L). As expected, IAV replication was significantly reduced in the cells treated with 17-AAG (Fig 8M). Together, these results indicate that PESP interact with HSP90AA1 to enhance its protein stability, which contributes to its function in promoting IAV replication via regulation of virus-induced autophagy.

## Discussion

It is well known that thousands of lncRNAs are differentially expressed upon viral infection. Some of these lncRNAs play crucial roles in the antiviral responses to IAV infection. However, others are hijacked by IAV to antagonize the host’s innate immune response or to assist with viral RNA synthesis [33]. Recent studies have discovered that many lncRNAs are not strictly non-coding, as they can encode micropeptides or small proteins through their sORF [27,28,34,35]. Despite the critical roles these molecules play in various biological processes, there is little information available about whether lncRNA-encoded small proteins/peptides function in influenza virus infection and replication. In the present study, we employed a joint analysis of Ribo-seq and RNA-seq, identified a new small protein, PESP, encoded by lncRNA PCBP1-AS1. The endogenous existence of PESP was confirmed by mass spectrometry and specific antibody detection.

LncRNAs can be highly induced by viral infection, with some serving as the host’s self-protective responses, and others being used by viruses for their replication and pathogenesis. For instance, the high expression of lincRNA VIN promotes viral production by facilitating the transcription and replication of influenza virus RNA genome [36]. LncRNA ACOD1 directly binds to glutamic-oxaloacetic transaminase (GOT2) in the cytoplasm, enhancing the catalytic activity of the enzyme and promoting the production of key metabolites required for viral replication, thereby facilitating production of virus in cells [37]. However, the MIR155HG gene plays a dual regulatory role in antiviral innate immune responses through encoding lncRNA-155 and miRNA-155-5p. LncRNA-155 can promote the production of virus-induced IFN-β by regulating the activation of interferon regulatory factor 3 (IRF3), while miRNA-155-5p enhances antiviral responses by promoting the activation of STAT1 [38]. LncRNA IFITM4P is significantly upregulated after IAV infection and acts as a ceRNA to inhibit the virus replication by regulating the mRNA levels of IFITM1, IFITM2, and IFITM3 [39]. Here, we found that IAV infection induced the expression of PCBP1-AS1 and PESP via interferon signaling. The initiation of interferon signaling requires RIG-I or MDA5 signaling through MAVS. Knockdown of RIG-I, MDA5, or MAVS led to a decrease in the expression of PCBP1-AS1. We noted an elevated expression of viral NP in cells with knockdown of RIG-I, MDA5, or MAVS compared to control cells, despite the reduced expression of PCBP1-AS1 that facilitates IAV replication. This may be attributed to the disruption of interferon signaling, which affects not only the expression of PCBP1-AS1 but also that of numerous antiviral ISGs. Moreover, using loss-of-function experiments, we demonstrated that PESP was essential for PCBP1-AS1 to exert its function in promoting IAV replication. This suggests an important mechanism by which the virus uses host lncRNA and its encoded small protein to promote replication.

PCBP1-AS1 belongs to the class of antisense lncRNAs whose transcription direction is opposite to that of adjacent coding genes. Studies have shown that antisense lncRNAs account for approximately 40% of the total cellular lncRNA population, making them the most abundant class of lncRNAs [40,41]. Some antisense lncRNAs form RNA-RNA duplexes with adjacent gene mRNAs, which enhances the stability of the adjacent gene mRNA and thereby upregulates the expression of the adjacent gene. For example, antisense lncRNA KRT7-AS forms an RNA-RNA duplex with KRT7, controlling the expression of KRT7 at the mRNA and post-transcriptional levels [42]. Other antisense lncRNAs achieve co-expression with adjacent coding genes by sharing promoters, thereby influencing the function of the adjacent coding genes [43]. In this study, we observed that PCBP1-AS1 and its encoded small protein PESP do not affect the expression level of the adjacent gene PCBP1 (S6 Fig), suggesting that there may not be an expression regulatory relationship between PCBP1-AS1/PESP and PCBP1.

Autophagy plays a complex role in the pathogenesis of viral diseases, acting as a double-edged sword. During viral infection, autophagy can either promote or inhibit viral replication [44]. In the case of influenza virus, previous studies revealed that cellular autophagy is involved in the viral replication [45]. Inducing autophagy through starvation or rapamycin treatment has been shown to increase the production of progeny viruses. Conversely, disrupting autophagy with siRNA and inhibitors reduces the production of progeny viruses [45]. Furthermore, the IAV NS1 protein has been found to stimulate autophagy by upregulating synthesis of HA and M2 [46]. Additionally, H5N1 influenza virus can trigger autophagy initiation by targeting the Akt-TSC2-mTOR signaling pathway. Inhibition of autophagy substantially increases the survival rate of mice and ameliorates the acute lung injury caused by H5N1 virus infection [47]. In this study, mRNA sequencing was performed in PESP-overexpressing cells and control cells, and GSEA results revealed that on top of the significantly enriched KEGG pathways appeared to be the ‘autophagy pathway’, indicating that PESP plays a role in the process of IAV-induced autophagy. Furthermore, we showed that PESP promotes IAV-induced autophagy and thereby enhanced replication of the virus. Interestingly, when inhibitors of different stages of autophagy were added to cells overexpressing PESP, it was observed that treatment with ULK1 inhibitor MRT68921 and PI3K inhibitor 3-MA, which work on the early stage to suppress autophagy, did not block the promoting effect of PESP on IAV replication. However, CQ treatment, which mainly targets the late stage of autophagy via suppression of lysosomal acidification, significantly diminished the upregulation of the virus production by PESP. Further experiments demonstrated that PESP promotes autophagy by upregulating the transcription of ATG7, an essential effector enzyme for elongation of autophagosomal vesicles that plays a critical role in regulating viral infection and pathogenesis [31]. These findings highlight the close relationship between PESP and influenza virus-induced autophagy. However, the precise molecular mechanism underlying regulation of ATG7 promoter activity by PESP remains to be further elucidated.

In addition, we identified HSP90AA1 as an interacting protein of PESP by immunoprecipitation and MS assay. The molecular chaperone HSP90AA1, also known as heat shock protein 90 alpha family class A member 1, is important for maintaining protein homeostasis and ensuring proper folding, stability, and function of various client proteins [48]. Previous studies have shown the involvement of HSP90AA1 in autophagy regulation. For example, HSP90AA1 has been shown to interact with the Cdc37 and ULK1, a key initiator of autophagy. This interaction promotes the activation of ULK1 and its subsequent phosphorylation, leading to the initiation of autophagy [49]. Influenza virus NP and M2 induce autophagy and an increase in HSP90AA1 expression. HSP90AA1 interacted with the virus PB2 protein, resulting in the increase of viral RNA synthesis and viral progeny production [50]. Additionally, the HA protein of influenza virus can bind to HSP90AA1, participating virus entry and inducing autophagy through the AKT-mTOR pathway [51]. Consistently, we found that the interaction between PESP and HSP90AA1 leads to stabilization of PESP, which contributes to subsequent enhancement of autophagy and increase of IAV replication. Our observations provide new evidence for the mechanism by which HSP90AA1 facilitates IAV-induced autophagy through stabilization of PESP and thereby increase of ATG7 expression.

Given the ability of IAV proteins, such as NP, M2, PB1-F2, NS1 and HA, to induce autophagy [46,50–52], we also explored potential crosstalk between PESP and viral protein-induced autophagy. Our results exhibited that co-transfection of plasmids expressing PESP and viral NP caused an increased accumulation of LC3-II compared to cells transfected with NP alone, suggesting that overexpression of PESP may enhance NP-induced autophagy, or PESP and NP may play synergistic roles in promoting autophagy. On the other hand, we observed that PESP overexpression had no significant impact on autophagy induced by the viral M2, PB1-F2, HA and NS1 proteins (S7 Fig).

Moreover, besides the role PCBP1-AS1 and PESP play in IAV infection, we also investigated the potential involvement of PCBP1-AS1 and PESP in other viral diseases. Our data demonstrated that both PCBP1-AS1 and PESP expression was upregulated during infections with SeV, MDRV, HSV-1, and PRV. We further evaluated the roles of PCBP1-AS1 and PESP in the viral infections, and observed that their overexpression in 293T cells facilitated the replication of MDRV, HSV-1 and PRV, but exerted an inhibitory effect on the replication of SeV (S8 Fig). These contrasting outcomes may be attributed to the divergent roles of autophagy in the replication of these viruses [53–56]. Nevertheless, further investigation is required to elucidate the mechanisms by which PCBP1-AS1 and PESP modulate the replication of different viruses.

In conclusion, frequent influenza virus infection and the infectious disease outbreaks remain a big global health concern. Fully understanding the molecular mechanisms of the viral infection and replication is crucial for epidemic prevention and control. This study provides evidence of the important role of a small protein encoded by lncRNA in IAV replication. It uncovers a new mechanism through which influenza virus exploits host factors to successfully replicate in cells. The identification and characterization of additional lncRNAs-encoded small proteins/peptides during influenza virus infection, as well as their interaction with the virus, will gain better insights into complicated mechanisms underlying the viral pathogenesis and aid in the discovery of antiviral drug targets.

## Materials and methods

### Cell lines and cell culture

The following cell lines were obtained from the American Type Culture Collection (ATCC) and cultured in Dulbecco’s Modified Eagle’s Medium (DMEM, Gibco, Grand Island, NY, USA) supplemented with 10% fetal bovine serum (FBS, Gibco) and 100 U/mL penicillin-streptomycin (Beyotime Biotechnology, Shanghai, China): A549 (human type II alveolar epithelial cells), 293T (human embryonic kidney cells), HeLa (human cervical adenocarcinoma cells), and MDCK (Madin-Darby canine kidney cells).

### Viruses and viral infection

The viruses used in this study were propagated as previously described [57,58]: Influenza virus A/WSN/33 (H1N1) (WSN), influenza virus A/PR/8/34 (H1N1) (PR8), and Sendai virus (SeV) in specific-pathogen-free (SPF) chicken embryos; Muscovy duck reovirus (MDRV) in duck embryo fibroblast cells; Herpes simplex virus 1 (HSV-1) in Vero cells; Pseudorabies virus (PRV) in PK15 cells.

For viral infection, the cells were infected with WSN, PR8, SeV, or HSV-1 at the indicated MOI. After 1 hour of adsorption at 37°C, the cells were washed with phosphate-buffered saline (PBS) and cultured in DMEM containing 2 μg/mL trypsin. Similarly, the cells were infected with MDRV or PRV at the indicated MOI. After 1 hour of adsorption at 37°C, the cells were washed with PBS and cultured in DMEM supplemented with 2% FBS.

### Plasmids and reagents

For plasmid construction, PCBP1-AS1, PCBP1-AS1-delPESP (in which the ORF for PESP was truncated) and PCBP1-AS1-mutPESP (in which the start codon of PESP was mutated) were cloned into the pNL-CMV vector. PESP was cloned into pEGFP-N1 vector and a modified pEGFP-N1 vector, in which the GFP start codon ATGGTG is mutated to ATTGTT (GFPmut), and PESP was fused in frame to the N-terminus of the GFPmut ORF. PESP was also cloned into the pLVX-3×FLAG vector and pcDNA3.1(-) vector with a 3×Flag tag at the C-terminus. The deletion mutants of PESP (Mut A, Δ7–22 aa; Mut B, Δ45–52 aa; Mut C, Δ89–109 aa) were inserted into pcDNA3.1(-) vector with a 3×Flag tag at the C-terminus.

MRT68921, 3-methyladenine (3-MA) and chloroquine (CQ) were purchased from MedChem Express (Shanghai, China). LPS was purchased from sigma (MO, USA). IFN-β and IL-6 were purchased from Peprotech (London, UK), and poly(I:C) was obtained from Invivogen (CA, USA). Anti-LC3 and anti-Flag were purchased from Sigma (MO, USA). Anti-HSP90AA1, anti-ATG7, and anti-HA were purchased from Proteintech (Wuhan, China). Anti-SQSTM1, anti-STAT3 and anti-p-STAT3 (Tyr705) were purchased from Cell Signaling Technology (MA, USA). Anti-β-actin was purchased from TransGen Biotech (Beijing, China). Anti-IAV PB2 was purchased from GeneTex (CA, USA), and NP was obtained as described previously [59]. The peptide (SSSLSMIIAKALKMAL) derived from PESP contained an N-terminal Tat peptide (YGRKKRRQRRR) for facilitating cellular penetration. It was labeled with 6-carboxyfluorescein (FAM) at the N-terminal and synthesized to a purity of 97% by GL Biochem (Shanghai, China). Peptides were dissolved in DMSO as a 20 mM stock solution and stored at −20°C.

### Cell stimulation

The PR8 genomic RNA (VG-RNA) was extracted from the purified virus particles using viral RNA kit (Omega, GA, USA) according to the manufacturer’s instructions. Viral RNA was isolated from PR8-infected A549 cells and control cellular RNA was prepared from uninfected A549 cells as previously described [59]. A549 cells were transfected with RNA or poly(I:C) using Lipofectamine 3000 (Invitrogen, CA, USA) for 8 h. For stimulation with cytokines, A549 cells were incubated with the recombinant IFN-β, IFN-λ1 or IL-6 (Peprotech, London, UK) for 6 h.

### RNA-seq and Ribo-seq

RNA-seq and Ribo-seq were performed by Novogene Co., Ltd (Beijing, China). For RNA-seq, the total RNA from A549 cells was extracted using Trizol reagent. The RNA integrity was then assessed using the RNA Nano 6000 Assay Kit of the Bioanalyzer 2100 system (Agilent Technologies, CA, USA). Following this, sequencing libraries were generated and their quality was evaluated on the Agilent Bioanalyzer 2100system. After library preparation, all samples were sequenced on an Illumina Novaseq platform.

For Ribo-seq, cells were lysed using lysis buffer with cycloheximide (50 mg/mL), and the cell lysate was treated with endoribonuclease RNase I to digest the RNA except ribosome-protected fragments (RPFs). After the RNA digestion, monosomes were isolated from the cell lysate using size-exclusion chromatography with MicroSpin S-400 HR columns. Following PAGE purification, both ends of the RPFs were phosphorylated and ligated with 5’ and 3’ adapters respectively. The RNA samples were then treated with rRNA depletion kit (Qiagen) to deplete rRNA contamination before PAGE purification of the relatively short (20~38 nt) RPFs. Then the fragments were reverse transcribed to the cDNAs and amplified by PCR to prepare a cDNA library. After library preparation and pooling of different samples, the samples were sequenced on an Illumina Hiseq 4000 platform.

### Preparation of anti-PESP antibody

Peptide synthesis and anti-PESP antibody preparation were performed by ABclonal Technology Co., Ltd. (Wuhan, China). Briefly, a KLH-coupled peptide RAPLSSRILLTDSP-Cys was synthesized, and polyclonal antibodies against the PESP peptide were obtained from inoculated rabbits. The immune sera were then affinity-purified using the immunizing peptide.

### Generation of cell lines

PESP knockout A549 cells were generated using CRISPR-Cas9 gene editing. The sgRNA oligo sequences targeting PESP (PESP-F, 5′-GACGTGGTCTTGTAACACAG-3′ and PESP-R, 5′-CTGTGTTACAAGACCACGTC-3′) were designed by the Cas9 design target tool (https://zlab.bio/guide-design-resources). The pairs of annealed oligos were separately introduced into pX458 after being digested with BbsI (Thermo Fisher Scientific). The subsequent steps of transfection, single cell screening and identification were performed as previously described [60].

Cells stably expressing PESP or empty vector (EV) were generated by infecting A549 cells with lentiviruses encoding these genes in pLVX-3×FLAG vector. Short hairpin RNA (shRNA)-based knockdown cell lines were generated by infection of A549 cells with lentiviruses expressing specific shRNAs in pSIH-H1-GFP vector as described previously [61]. The sequences used in the shRNAs targeting PCBP1-AS1 and PESP were as described in S1 Table.

### Immunoprecipitation, mass spectrometry and Western blotting

Preparation of cell extracts and immunoprecipitation were performed as previously described [62]. Briefly, cell extracts were immunoprecipitated overnight at 4°C with indicated antibodies. Immunoprecipitates were washed three times with lysis buffer and then separated by SDS-PAGE. Gel pieces cut from SDS-PAGE were transferred to Shanghai Hoogen Biotechnology Company for mass spectrometry analysis. For Western blotting, cell lysates were separated by SDS-PAGE and transferred onto the nitrocellulose membrane, and probed with antibodies as indicated.

### Immunofluorescence assay

Immunofluorescence assay was performed as described previously [63,64]. Plasmid-transfected cells were fixed with 4% paraformaldehyde for 30 min at room temperature and then permeabilized with 0.1% Triton-100 for 4 min. After washing, the cells were blocked with 1% BSA-PBS for 30 min at room temperature. Then, the cells were incubated with anti-Flag antibody for 2 h at 37°C, followed by incubation with secondary antibody (CoraLite594-conjugated Goat Anti-Mouse IgG, Proteintech, Wuhan, China) for 1 h at 37°C. After washing, the nuclei were stained with DAPI for 5 minutes. The cells were observed using a Zeiss LSM 880 confocal microscope (Zeiss, Jena, Germany). Images were processed with ZEN 2.3 Blue Edition software (Zeiss, Jena, Germany).

### Dual-luciferase reporter assay

The ATG7 promoter (from −1398 to −227) was amplified as previously reported [65], and was inserted into the firefly luciferase reporter vector pGL3-Basic. 293T cells were seeded in 24-well culture plates, and were transfected with the luciferase report plasmid, pRL-TK together with the plasmid encoding PESP, Mut A (the deletion mutant of PESP) or the control plasmid. After 36 h of transfection, the cells were lysed, followed by dual-luciferase activity assays using a dual-luciferase reporter assay kit (Promega, WI, USA) according to the manufacturer’s instructions. Luciferase activity was normalized to that of Renilla luciferase activity.

### Cell counting kit-8 (CCK-8) assay

WT and PESP-KO A549 cells were seeded in 100 μL of growth medium at a density of 2 × 10^3^ cells per well in 96-well plates. Following an overnight incubation, cell proliferation was evaluated by CCK-8 assay. At the indicated time points, 10 μL CCK-8 solution (Beyotime Biotechnology, Shanghai, China) was added per well and incubated for 2 h at 37°C. Absorbance was determined at 450 nm using a microplate reader (Tecan Infinite M200 Pro, Switzerland).

### RNA preparation, RT-PCR, and qRT-PCR

Total RNA was extracted from cells using TRNzol reagent (TIANGEN, Beijing, China). cDNA was synthesized by a HiScript III 1st Strand cDNA Synthesis Kit (Vazyme, Nanjing, China). Then, the cDNA was used for PCR or quantitative real-time PCR (qRT-PCR) by Taq DNA polymerase (GenStar, Beijing, China) or SYBR Green Master Mix (Vazyme, Nanjing, China). The primer sequences are available upon request. GAPDH was used as a reference housekeeping gene for internal standardization. The data of qPCR analysis were shown in normalized ratios which was auto-calculated using DDCT method by LightCycler system (Roche, Switzerland).

### Hemagglutination assay and plaque-forming assay

Hemagglutination (HA) assay and plaque assay were performed as previously described [57,59]. Briefly, for HA assay, the supernatants were diluted with PBS and mixed with an equal volume of 0.5% chicken erythrocytes. Then, the viral titers were counted 20 min later. For plaque assay, MDCK cells infected with serial dilutions of the supernatants of cell cultures were washed with PBS and overlaid with α-minimal essential medium containing 1.5% low-melting-point agarose (Promega, WI, USA) and 2 μg/mL TPCK (tolylsulfonyl phenylalanyl chloromethyl ketone)-treated trypsin (Sigma-Aldrich, MO, USA). After incubation for 72 h at 37°C, plaques were stained and counted.

### Statistical analysis

Comparison between groups was made using Student’s *t* test. Data represent the mean ± SEM from three independent experiments. Differences were considered statistically significant with *p* < 0.05.

## Supporting information

S1 Fig Endogenous peptides of PESP are identified by MS. (A, B) Two unique peptides of PESP in A549 cell lysates immunoprecipitated with PESP-specific antibody were identified by mass spectrometry (MS).
(TIF)

S2 Fig The protein expression of PESP is induced by multiple viral infections. (A-D) The expression of PESP in 293T cells infected with SeV (A), MDRV (B), HSV-1 (C), and PRV (D) was examined by Western blotting. n = 3.
(TIF)

S3 Fig Poly(I:C) and interferons, but not IL-6 and LPS, induce the expression of PCBP1-AS1. (A) A549 cells were transfected with various concentrations of poly(I:C) for 8 h. Then the cells were harvested, and the expression of PCBP1-AS1 was examined by RT-PCR. (B) A549 cells were treated with IFN-β at indicated concentrations for 6 h. The expression of PCBP1-AS1 was examined by RT-PCR. (C) Relative levels of PESP in Fig 3H were quantitated by densitometry and normalized to β-actin levels. (D) A549 cells were treated with IFN-λ1 at indicated concentrations for 6 h. The expression of PCBP1-AS1 was examined by RT-PCR. (E) A549 cells were treated with IL-6 at the indicated concentrations for 6 h. The expression of PCBP1-AS1 was detected by RT-PCR. (F) A549 cells were incubated with LPS at the indicated concentrations for 6 h. The expression of PCBP1-AS1 was determined by RT-PCR. Data are shown as means ± SD; n = 3; *p< 0.05, **p< 0.01.
(TIF)

S4 Fig Generation of PESP knockout cell lines and role of PESP in influenza virus replication. (A) PESP knockout A549 cells were generated using CRISPR-Cas9-mediated gene editing. The mutation of PESP in knockout cell lines was verified by sequencing analysis. (B) CCK-8 assay was performed to measure the proliferation of WT and PESP-knockout A549 cells at the indicated time. (C) 293T cells were transfected with EV, PESP, PCBP1-AS1 or PCBP1-AS1-delPESP, followed by infection with PR8 virus (MOI = 0.5) for 16 h. The mRNA levels of viral NP and NS1 in the cells were examined by RT-PCR. (D, E) 293T cells were transfected with EV, PESP, PCBP1-AS1 or PCBP1-AS1-mutPESP, followed by infection with PR8 (MOI = 0.5) for 16 h. The mRNA levels of viral NP in the cells were examined by RT-PCR (D) and qRT-PCR (E). Data are shown as means ± SD; n = 3; *p< 0.05, **p< 0.01, and ns represents no significance.
(TIF)

S5 Fig PESP upregulates the expression of ATG7 in 293T cells. PESP-overexpressing 293T cells and control cells were infected with PR8 virus (MOI = 0.5) for 12 h, and then the cells were harvested and analyzed by Western blotting with the indicated antibodies. n = 3.
(TIF)

S6 Fig Overexpression of PCBP1-AS1 and PESP have no effects on the expression of PCBP1. (A, B) A549 cells overexpressing PCBP1-AS1 (A) or PESP (B) and control cells were infected with or without PR8 virus (MOI = 1) for 12 h, and the mRNA levels of PCBP1 were examined by qRT-PCR. Data are represented as mean ± SD; n = 3; ns represents no significance.
(TIF)

S7 Fig Role of PESP in autophagy induced by IAV proteins. (A, B) 293T cells were co-transfected EV or PESP plasmid together with plasmid expressing PR8 NP for 24 h. The cells were harvested and analyzed by Western blotting with the indicated antibodies (A). Relative levels of LC3-II in (A) were quantitated by densitometry and normalized to β-actin levels (B). (C-F) 293T cells were co-transfected EV or PESP plasmid together with plasmids expressing PR8 M2 (C), PB1-F2 (D), NS1 (E) or HA (F) for 24 h. The cells were harvested and analyzed by Western blotting with the indicated antibodies. Data are shown as means ± SD; n = 3; *p< 0.05.
(TIF)

S8 Fig Role of PCBP1-AS1 and PESP in the replication of SeV, MDRV, HSV-1 and PRV. 293T cells were transfected with EV or plasmids expressing PESP or PCBP1-AS1 for 24 h, followed by infection with SeV (A), MDRV (B), HSV-1(C), or PRV (D). The expression of SeV-NP, MDRV-P10, HSV-UL30, and PRV-gE in these cells was examined by qRT-PCR. Data are represented as mean ± SD; n = 3; *p< 0.05, **p< 0.01.
(TIF)

S1 Table Sequences of shRNAs and siRNA used in this study. (XLSX)
