# Supplementary figures and images for: A small protein encoded by PCBP1-AS1 is identified as a key regulator of influenza virus replication via enhancing autophagy
Source: PLoS Pathog. 2024 Aug 13;20(8):e1012461. doi: 10.1371/journal.ppat.1012461 (PMC11343454; doi:10.1371/journal.ppat.1012461)

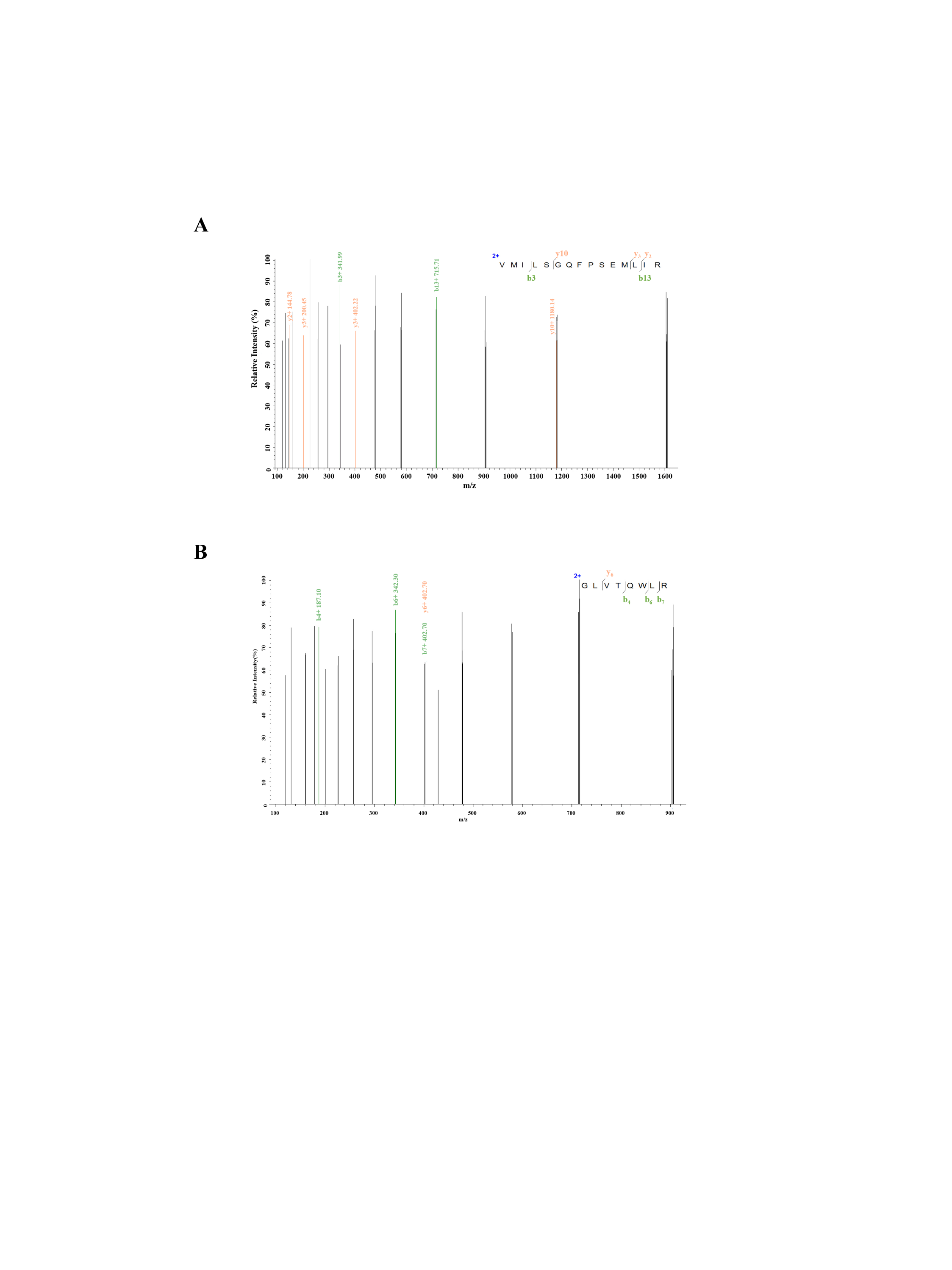

Supplement: S1 Fig — (A, B) Two unique peptides of PESP in A549 cell lysates immunoprecipitated with PESP-specific antibody were identified by mass spectrometry (MS). (TIF) [file ppat.1012461.s001.tif]

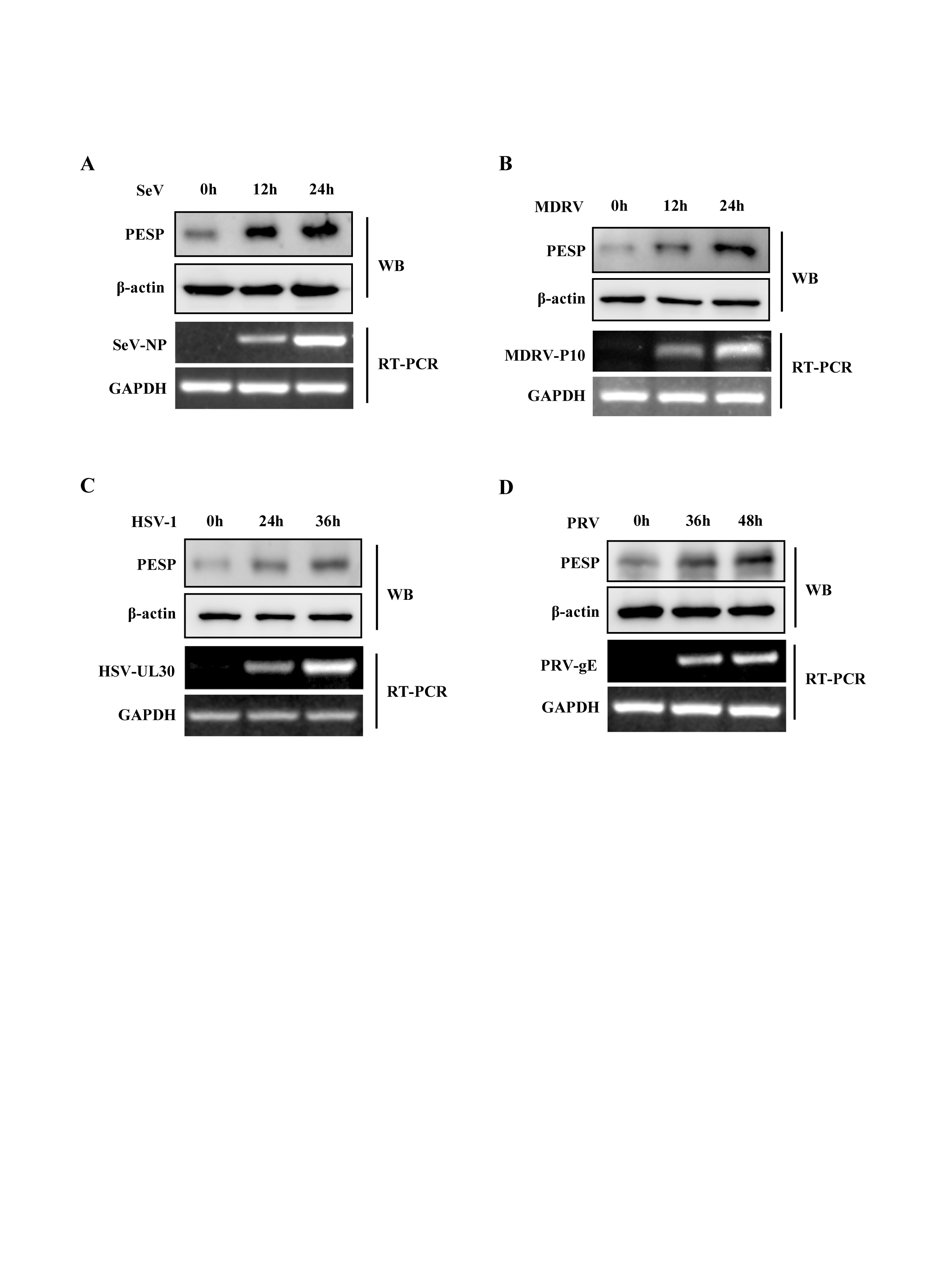

Supplement: S2 Fig — (A-D) The expression of PESP in 293T cells infected with SeV (A), MDRV (B), HSV-1 (C), and PRV (D) was examined by Western blotting. n = 3. (TIF) [file ppat.1012461.s002.tif]

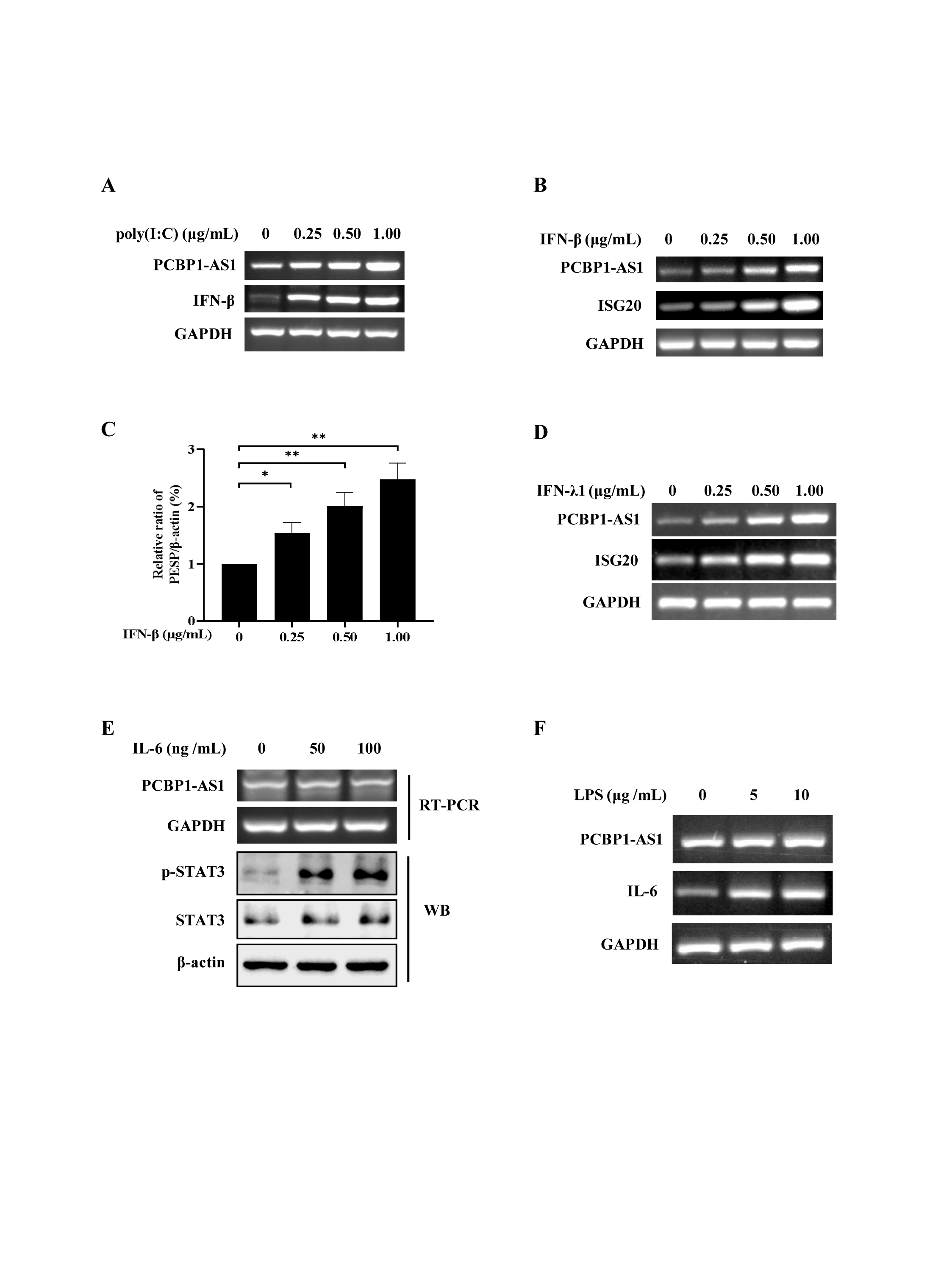

Supplement: S3 Fig — (A) A549 cells were transfected with various concentrations of poly(I:C) for 8 h. Then the cells were harvested, and the expression of PCBP1-AS1 was examined by RT-PCR. (B) A549 cells were treated with IFN-β at indicated concentrations for 6 h. The expression of PCBP1-AS1 was examined by RT-PCR. (C) Relative levels of PESP in Fig 3H were quantitated by densitometry and normalized to β-actin levels. (D) A549 cells were treated with IFN-λ1 at indicated concentrations for 6 h. The expression of PCBP1-AS1 was examined by RT-PCR. (E) A549 cells were treated with IL-6 at the indicated concentrations for 6 h. The expression of PCBP1-AS1 was detected by RT-PCR. (F) A549 cells were incubated with LPS at the indicated concentrations for 6 h. The expression of PCBP1-AS1 was determined by RT-PCR. Data are shown as means ± SD; n = 3; *p< 0.05, **p< 0.01. (TIF) [file ppat.1012461.s003.tif]

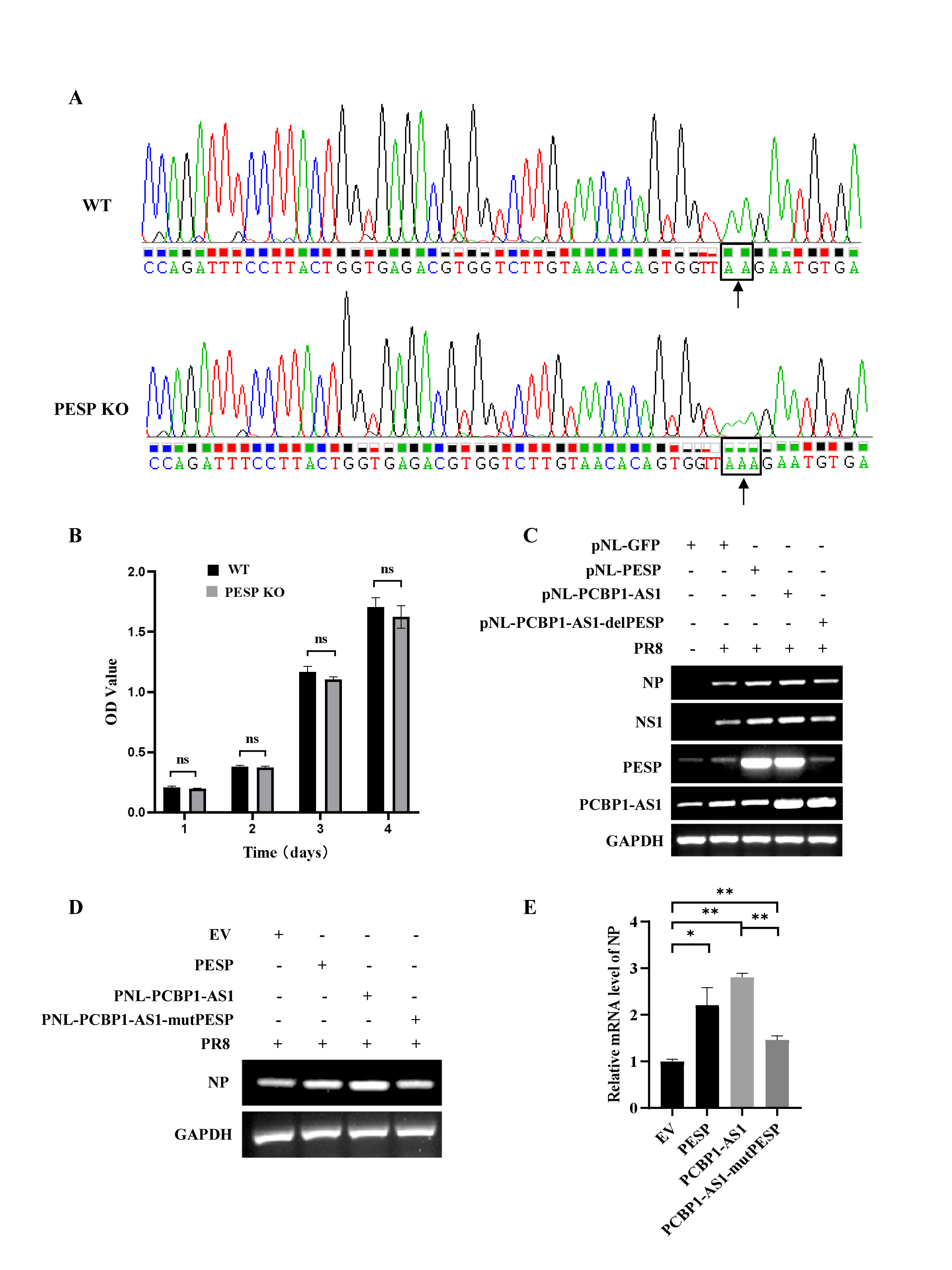

Supplement: S4 Fig — (A) PESP knockout A549 cells were generated using CRISPR-Cas9-mediated gene editing. The mutation of PESP in knockout cell lines was verified by sequencing analysis. (B) CCK-8 assay was performed to measure the proliferation of WT and PESP-knockout A549 cells at the indicated time. (C) 293T cells were transfected with EV, PESP, PCBP1-AS1 or PCBP1-AS1-delPESP, followed by infection with PR8 virus (MOI = 0.5) for 16 h. The mRNA levels of viral NP and NS1 in the cells were examined by RT-PCR. (D, E) 293T cells were transfected with EV, PESP, PCBP1-AS1 or PCBP1-AS1-mutPESP, followed by infection with PR8 (MOI = 0.5) for 16 h. The mRNA levels of viral NP in the cells were examined by RT-PCR (D) and qRT-PCR (E). Data are shown as means ± SD; n = 3; *p< 0.05, **p< 0.01, and ns represents no significance. (TIF) [file ppat.1012461.s004.tif]

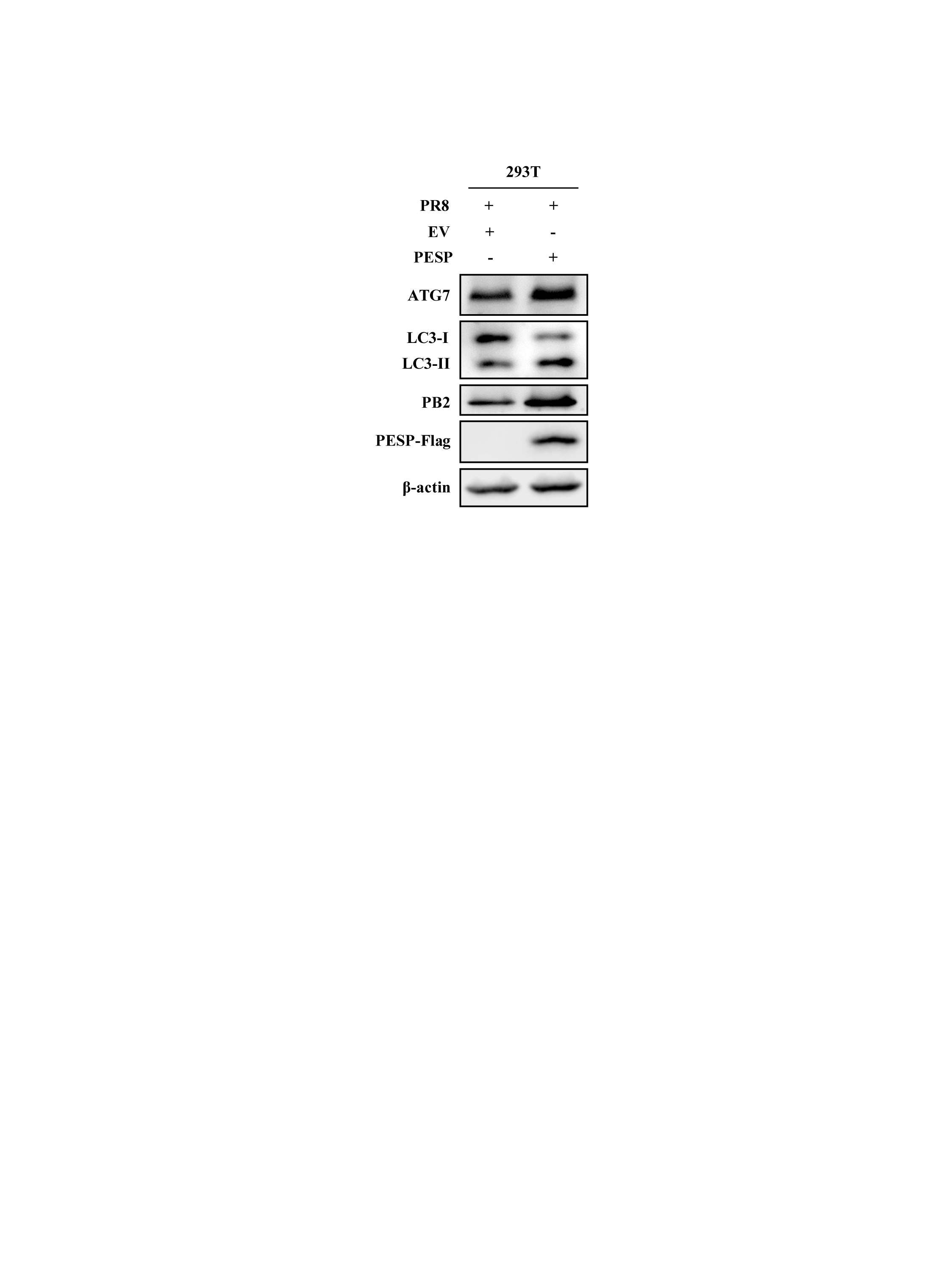

Supplement: S5 Fig — PESP-overexpressing 293T cells and control cells were infected with PR8 virus (MOI = 0.5) for 12 h, and then the cells were harvested and analyzed by Western blotting with the indicated antibodies. n = 3. (TIF) [file ppat.1012461.s005.tif]

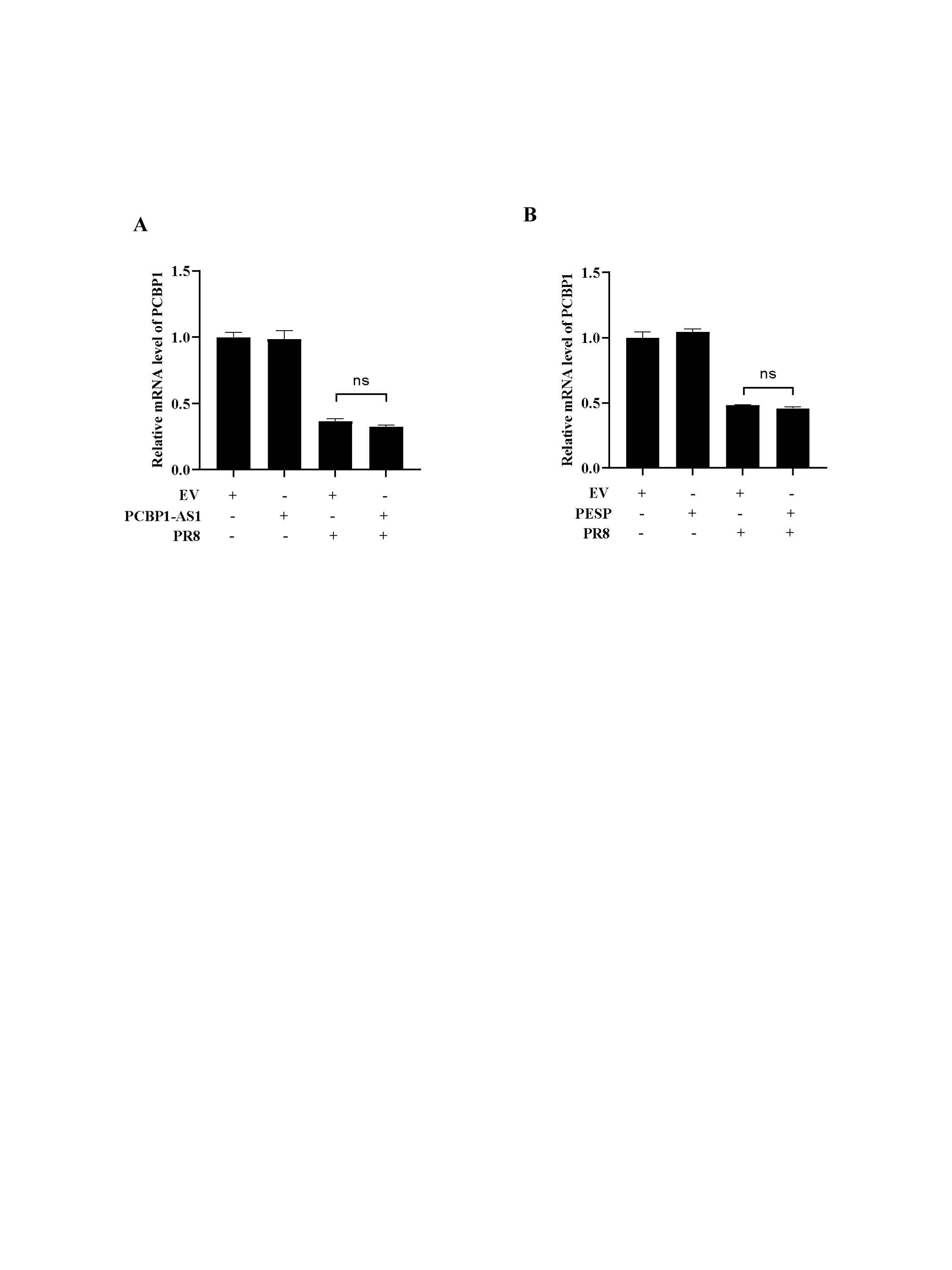

Supplement: S6 Fig — (A, B) A549 cells overexpressing PCBP1-AS1 (A) or PESP (B) and control cells were infected with or without PR8 virus (MOI = 1) for 12 h, and the mRNA levels of PCBP1 were examined by qRT-PCR. Data are represented as mean ± SD; n = 3; ns represents no significance. (TIF) [file ppat.1012461.s006.tif]

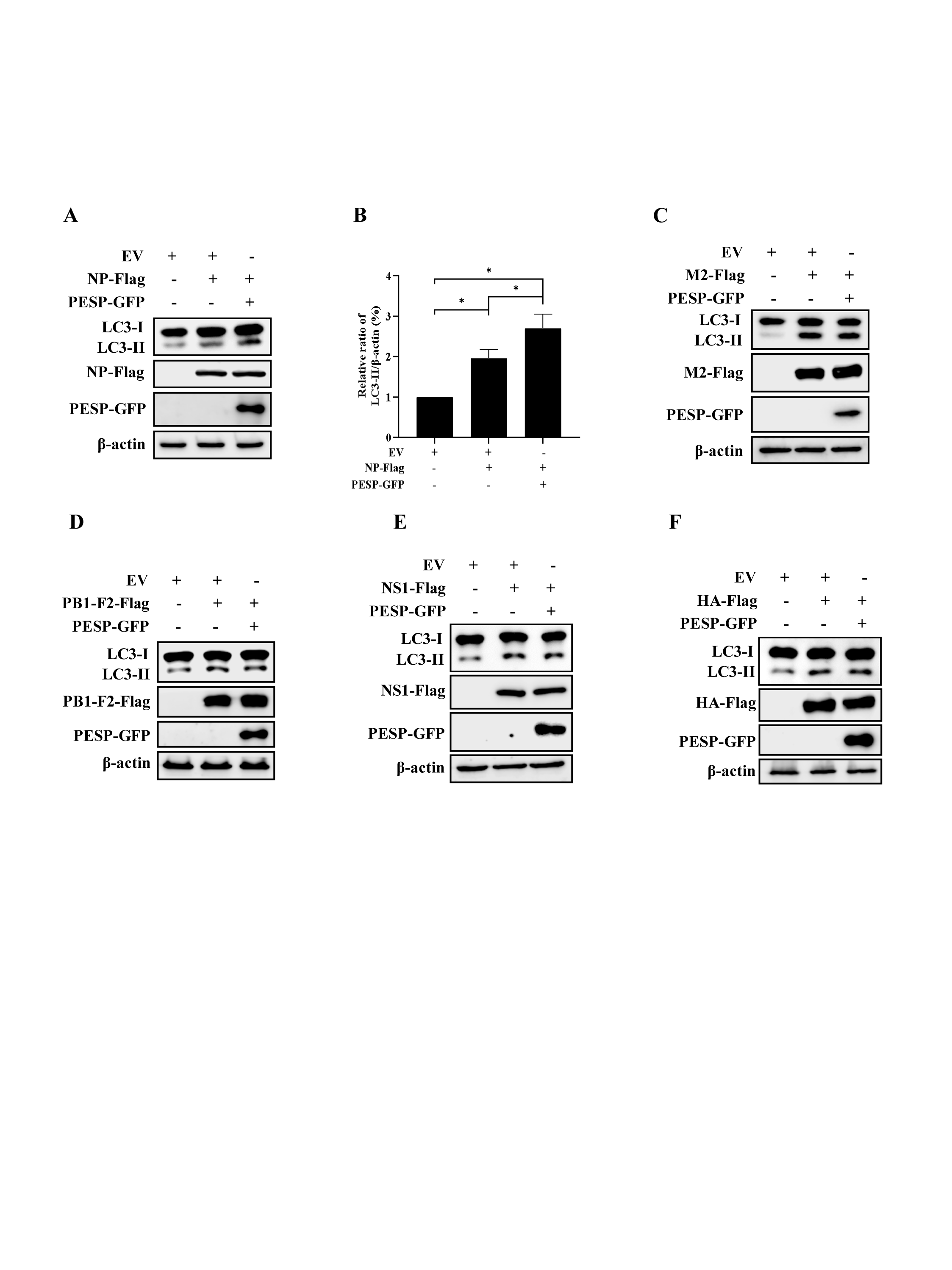

Supplement: S7 Fig — (A, B) 293T cells were co-transfected EV or PESP plasmid together with plasmid expressing PR8 NP for 24 h. The cells were harvested and analyzed by Western blotting with the indicated antibodies (A). Relative levels of LC3-II in (A) were quantitated by densitometry and normalized to β-actin levels (B). (C-F) 293T cells were co-transfected EV or PESP plasmid together with plasmids expressing PR8 M2 (C), PB1-F2 (D), NS1 (E) or HA (F) for 24 h. The cells were harvested and analyzed by Western blotting with the indicated antibodies. Data are shown as means ± SD; n = 3; *p< 0.05. (TIF) [file ppat.1012461.s007.tif]

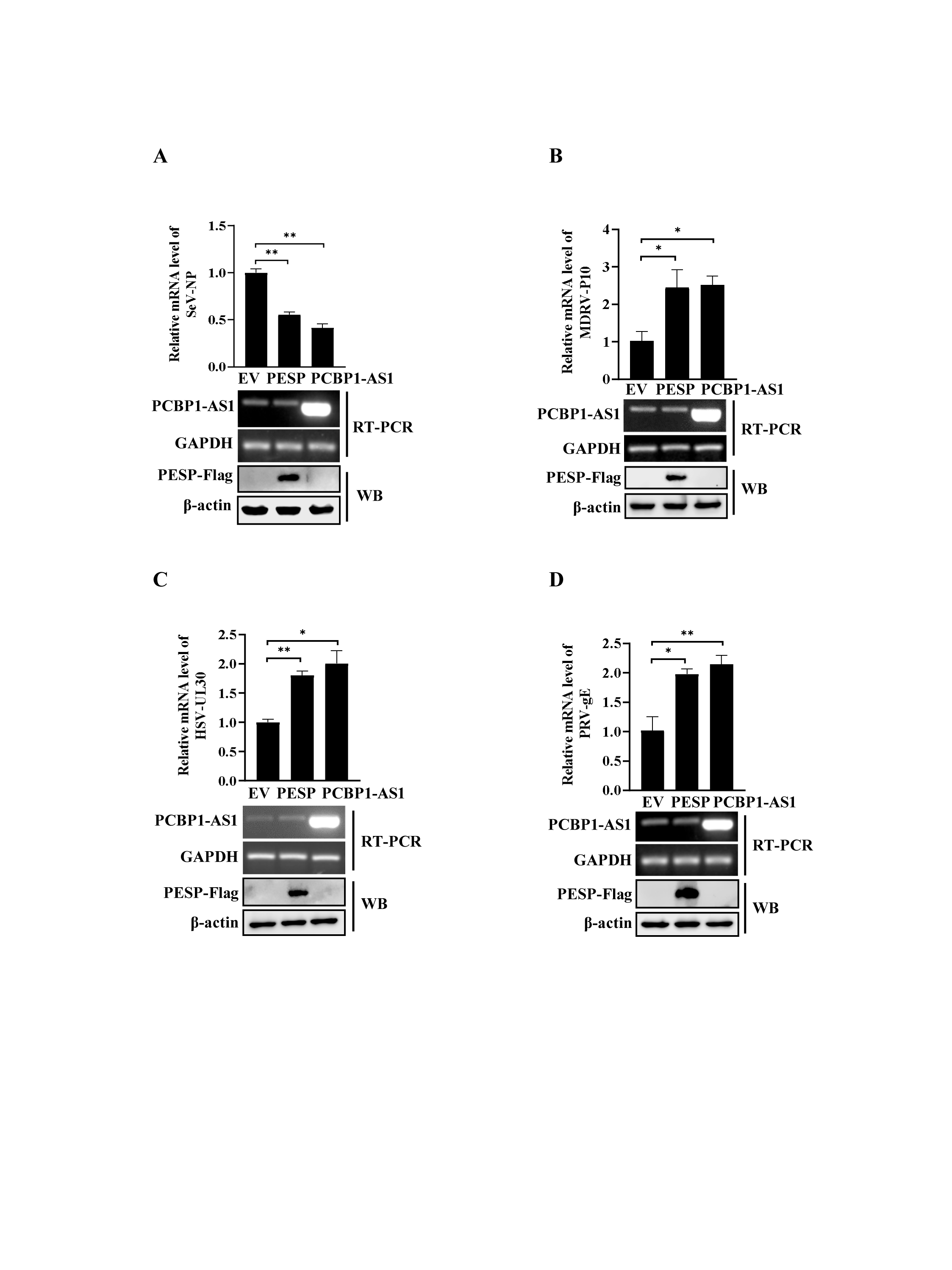

Supplement: S8 Fig — 293T cells were transfected with EV or plasmids expressing PESP or PCBP1-AS1 for 24 h, followed by infection with SeV (A), MDRV (B), HSV-1(C), or PRV (D). The expression of SeV-NP, MDRV-P10, HSV-UL30, and PRV-gE in these cells was examined by qRT-PCR. Data are represented as mean ± SD; n = 3; *p< 0.05, **p< 0.01. (TIF) [file ppat.1012461.s008.tif]
